# White Matter Integrity Declined Over 6-Months, but Dance Intervention Improved Integrity of the Fornix of Older Adults

**DOI:** 10.3389/fnagi.2017.00059

**Published:** 2017-03-16

**Authors:** Agnieszka Z. Burzynska, Yuqin Jiao, Anya M. Knecht, Jason Fanning, Elizabeth A. Awick, Tammy Chen, Neha Gothe, Michelle W. Voss, Edward McAuley, Arthur F. Kramer

**Affiliations:** ^1^Department of Human Development and Family Studies, Molecular, Cellular and Integrative Neurosciences, Colorado State UniversityFort Collins, CO, USA; ^2^The Beckman Institute for Advanced Science and Technology at the University of IllinoisUrbana, IL, USA; ^3^Department of Kinesiology and Community Health, University of IllinoisUrbana, IL, USA; ^4^Division of Kinesiology, Health and Sport Studies, Wayne State UniversityDetroit, MI, USA; ^5^Psychological and Brain Sciences, University of IowaIowa City, IO, USA; ^6^Senior Vice Provost for Research and Graduate Education, Northeastern UniversityBoston, MA, USA

**Keywords:** DTI, diffusion, randomized clinical trial, fractional anisotropy, processing speed, physical activity, fitness, brain

## Abstract

Degeneration of cerebral white matter (WM), or structural disconnection, is one of the major neural mechanisms driving age-related decline in cognitive functions, such as processing speed. Past cross-sectional studies have demonstrated beneficial effects of greater cardiorespiratory fitness, physical activity, cognitive training, social engagement, and nutrition on cognitive functioning and brain health in aging. Here, we collected diffusion magnetic resonance (MRI) imaging data from 174 older (age 60–79) adults to study the effects of 6-months lifestyle interventions on WM integrity. Healthy but low-active participants were randomized into Dance, Walking, Walking + Nutrition, and Active Control (stretching and toning) intervention groups (NCT01472744 on ClinicalTrials.gov). Only in the fornix there was a time × intervention group interaction of change in WM integrity: integrity declined over 6 months in all groups but increased in the Dance group. Integrity in the fornix at baseline was associated with better processing speed, however, change in fornix integrity did not correlate with change in processing speed. Next, we observed a decline in WM integrity across the majority of brain regions in all participants, regardless of the intervention group. This suggests that the aging of the brain is detectable on the scale of 6-months, which highlights the urgency of finding effective interventions to slow down this process. Magnitude of WM decline increased with age and decline in prefrontal WM was of lesser magnitude in older adults spending less time sedentary and more engaging in moderate-to-vigorous physical activity. In addition, our findings support the anterior-to-posterior gradient of greater-to-lesser decline, but only in the in the corpus callosum. Together, our findings suggest that combining physical, cognitive, and social engagement (dance) may help maintain or improve WM health and more physically active lifestyle is associated with slower WM decline. This study emphasizes the importance of a physically active and socially engaging lifestyle among aging adults.

## Introduction

Disruption of (WM) microstructure—degeneration or loss of axons and myelin—is considered one of the primary mechanisms underlying age-related cognitive slowing and memory decline (Gunning-Dixon and Raz, 2000; Madden et al., 2012). Therefore, preventing age-related “structural disconnection” (Raz and Rodrigue, 2006) or improving WM integrity is key in preserving cognitive performance necessary for independent functioning in older individuals.

WM microstructure can be studied non-invasively with diffusion magnetic resonance imaging (MRI). Diffusion imaging provides voxel-wise estimation of magnitude and directionality of water diffusion in WM. Fractional anisotropy (FA), is a measure of the directional dependence of diffusion (Basser, 1995), and reflects fiber orientation, density and coherence within a voxel (Beaulieu, 2002). Lowered FA has been observed in various conditions in which loss of fiber integrity occurs (Beaulieu, 2002), such as Alzheimer's disease (Medina et al., 2006). Radial diffusivity (RD) represents diffusivity perpendicular to the main fiber direction (Basser, 1995; Song et al., 2002). Increases in RD have been linked to degeneration or loss of myelin (Song et al., 2003, 2005). Axial diffusivity (AD) represents diffusion parallel to the axon fibers and is related to axonal integrity (Basser, 1995; Song et al., 2002). Finally, mean diffusivity (MD) reflects the magnitude of total water diffusion within a voxel, which depends on the density of physical obstructions such as cellular membranes (Beaulieu, 2002; Sen and Basser, 2005). Increased MD, paralleled by increases in both RD and AD, was observed in conditions of WM degeneration (Beaulieu et al., 1996; Beaulieu, 2002; Concha et al., 2006).

To date, numerous neuroimaging studies described age-related differences in WM properties using cross-sectional comparisons (Burzynska et al., 2010; Madden et al., 2012). There are, however, two critical obstacles in understanding the age-related changes in WM and, subsequently, in slowing down or reversing these age-related changes in the human brain. First, there are still few studies describing age-related change in WM integrity in a longitudinal design. Specifically, there are only five studies that described changes in WM over time and across numerous WM regions or tracts^1^. Sexton et al. (2014) and Storsve et al. (2016) followed 203 adults between 20 and 84 years of age over on average 3.5 years. They found extensive and overlapping, significant annual decreases in FA, paralleled by increases in RD, AD, and MD. Rieckmann et al. (2016) followed up 108 older adults over on average 2.6 years and found significant declines in FA and increases in RD, AD, and MD. Bender et al. (2016b) found changes in FA and RD over periods of time of 1 to 7 years in healthy adults of age 50–84. Barrick et al. (2010) observed significant decline in FA in healthy adults 50–90 years old over 2 years.

Some of these studies reported acceleration of microstructural decline in older age (Sexton et al., 2014; Bender et al., 2016b; Storsve et al., 2016), but other did not (Barrick et al., 2010). Some argued the superior-to-inferior gradient of greater-to-lesser decline (Sexton et al., 2014; Storsve et al., 2016), while longitudinal data (Barrick et al., 2010) did not support “last-in-first-out” hypothesis of anterior-to-posterior decline suggested in cross-sectional studies (Bartzokis et al., 2010).

Together, these studies show consistent decline in WM integrity represented by increases in RD, AD, and MD, and decreases in FA. However, there is no consensus on the spatial gradient of decline and WM decline has not been observed over periods shorter than a year. Knowing short-term dynamics of WM decline would be useful in assessing the outcomes of typically short-term interventions (months) as well as in differentiating between normal and abnormal speed of decline in patients presenting first cognitive symptoms. Finally, there is little evidence for the ability to improve WM integrity in older adults. Cross-sectional studies suggest that lifestyle factors such as physical activity (PA) and cardiorespiratory fitness (CRF) are protective against cognitive and neural decline. For example, we have shown that greater PA and CRF are associated with greater WM integrity (Burzynska et al., 2014; Oberlin et al., 2016) and that older aerobically trained athletes have greater brain structural integrity and cognitive performance than their sedentary low-fit peers (Tseng et al., 2013; Burzynska et al., 2015; Young et al., 2016). However, a recent meta-analysis showed only modest cross-sectional effects of CRF and aerobic PA on WM in aging (Sexton et al., 2016). The longitudinal evidence for positive effect of exercise on WM is still very scarce. Voss et al. (2010) demonstrated in 70 adults (55–80 years old) that increases in CRF as a result of 1-year the aerobic walking intervention was associated with fronto-temporal increase in FA and enhanced short-term memory. However, there was no difference between the walking and the active control group (stretching and toning) in their changes of WM integrity over 1-year.

In the current study we address these two critical limitations of the existing studies: short-term dynamics in WM change in different diffusivity parameters, and the effects of lifestyle interventions to improve WM integrity in aging.

To this aim, we collected diffusion, cognitive, CRF and PA data from 174 healthy, non-demented (MMSE>26) adults 60–79 years old at baseline^2^, and after a 6-months lifestyle intervention (randomized clinical trial, NCT01472744 on ClinicalTrials.gov). The interventions included aerobic exercise (Walking) and an Active Control group (stretching and toning, not aimed to increase CRF). In addition, we included a group that combined aerobic PA, cognitive, and social stimulation (Dance), and an aerobic Walking that also received a nutritional supplement (Walking + Nutrition).

We expected to observe a time × group interaction, with Walking and Dance groups showing maintenance or increase in WM integrity as compared to the decline in the Active Control group. We expected to observe this effect especially in the frontal and temporal regions (Colcombe et al., 2006; Voss et al., 2010). Next, we expected to observe declines in FA and increases in MD, RD, and AD across the WM and that this decline will be accelerated in the oldest, more sedentary, less active, and less fit (lower CRF) adults. However, given the shorter time scale, we expected these changes to be of smaller magnitude and more spatially restricted than in the existing studies with time lags greater than a year. Finally, we expected that change in FA in the Walking or Dance groups would be behaviorally relevant, i.e., be related to change in cognitive performance, especially in the speed and memory domains (Lövdén et al., 2014; Wang et al., 2015; Bender et al., 2016a) as compared to crystallized and fluid abilities (Virginia Cognitive Aging Project Battery; Salthouse and Ferrer-Caja, 2003; Salthouse, 2004, 2005, 2010)^3^.

## Methods

### Participants

The University of Illinois institutional review board approved this study, written informed consent was obtained from all participants and the study was performed in accordance with the 1964 Declaration of Helsinki. The sample was recruited to participate in a randomized controlled exercise trial (“Influence of Fitness on Brain and Cognition II” at ClinicalTrials.gov, clinical study identifier NCT01472744). Healthy, low active older adults were recruited in the Champaign county area to participate in a series of neuroimaging, cognitive, and cardiorespiratory testing, before and after a 6-months aerobic exercise intervention program. Of the 1,119 participants recruited, 247 (*n* = 169 women) met inclusion criteria and agreed to enroll in the study (See Supplementary Material 1 for subject flow). Eligible participants met the following criteria: (1) were between the ages of 60 and 80 years old; (2) were free from psychiatric and neurological illness and had no history of stroke or transient ischemic attack; (3) scored <10 on the geriatric depression scale (GDS-15); (4) scored ≥75% right-handedness on the Edinburgh Handedness Questionnaire; (5) demonstrated normal or corrected-to-normal vision of at least 20/40 and no color blindness; (6) cleared for suitability in the MRI environment; that is, no metallic implants that could interfere with the magnetic field or cause injury, no claustrophobia, and no history of head trauma; (7) reported to have participated in no more than two moderate bouts of exercise per week within the past 6-months; (8) were not taking medication for cardiovascular disease (e.g., beta blocker, diuretics), neurological, or psychiatric conditions (e.g., antidepressant, neuroleptic, anxiolytic). The sample contained more females because fewer older males met the above inclusion criteria or showed willingness to participate in the study. After the baseline measurement, the participants were randomized using a computer data management system and baseline-adaptive randomization scheme (Begg and Iglewicz, 1980) into four intervention group (Walking *n* = 54, Walking + Nutrition *n* = 54, Dance *n* = 69, Active Control *n* = 70). Randomization was stratified by gender and age. Neither self-reported nor objectively measured PA was used as a randomization criterion.

The timeline for data collection was as follows: (1) Pre-Screening Interview and Mock MRI session; (2) Neuropsychological assessments (Virginia Cognitive Aging Battery but also spatial working memory task, task switching, not discussed here but described in the Supplementary Material 2); (3) Street crossing assessment (data presented elsewhere); (4) MRI session; (5) Treadmill test (CRF testing). We aimed to complete the above sessions within 3 weeks, but due to participant's availability it took longer for several subjects. All tests were completed at least 1 day before intervention onset. Sessions 2–5 were repeated after 6-months intervention.

### Interventions

Following baseline cognitive and cardiorespiratory assessment, participants were randomized into one of the four intervention groups, taking into account equal distributions of age and gender: Dance, Walking, Walking + Nutrition, and Active Control. All participants attended supervised, 1-h sessions three times per week for 6-months. **Dance:** This intervention was designed to improve physical fitness as well as aspects of cognition necessary for learning complex social dance sequences in a socially engaging environment. Sessions were conducted in an appropriate dance space and were taught by experienced dance instructors. The choreographed dance combinations became progressively more challenging over the course of the 6-months program. Group social dance styles were selected (i.e., Contra and English Country dancing) to minimize lead-follow roles. Instead, these social dances required participants to move between partners during each dance. Each participant learned and alternated between two roles for each dance, increasing the cognitive challenge. **Walking:** This intervention was designed to increase CRF through brisk walking. Research staff supervised all walking sessions. Frequent assessment of heart rate, using either palpation or Polar Heart Rate Monitors, and rating of perceived exertion ensured that participants' exercise intensity was performed at the prescribed level. Exercise logs were completed after each exercise session to assess exercise frequency, intensity (RPE) and enjoyment levels. **Walking** + **Nutrition:** The Walking + Nutrition condition engaged in the same protocol as those in the walking condition. Additionally, they ingested a daily supplement supplied by Abbot Nutrition that contained beta-alanine. Beta alanine is thought to promote an increase in lean muscle mass (Zoeller et al., 2007), thereby enhancing the effect of increased CRF on brain health to boost the effect of increased CRF on brain health. Research staff supervised all walking sessions. Frequent assessment of heart rate, using either palpation or Polar Heart Rate Monitors, and rating of perceived exertion ensured that participants' exercise intensity was performed at the prescribed level. Exercise logs were completed after each exercise session. Participants were instructed to take the supplement drink daily, which was a liquid, milk-based formula supplied by Abbott Nutrition. **Active Control:** This intervention served as the active control group to account for the social engagement in the other interventions. A trained exercise specialist at a facility on the University of Illinois campus conducted all strength and balance sessions. This program focused on improving strength, stretching and stability for the whole body and was specifically designed for individuals 60 years of age and older. The program includes non-aerobic stretches, simple strength exercises, and basic balancing activities for all the large muscle groups. Each stretch was gently held to a point of slight tension but not pain for approximately 20–30 s. Each stretching and toning session included a 10–15 min warm-up and cool-down and 30–45 min of the above described stretching and toning exercises. Participants completed exercise logs on a weekly basis. The intervention was conducted in four waves from October 2011 to November 2014.

### Diffusion tensor imaging (DTI)

Diffusion-weighted images were acquired on a 3T Siemens Trio Tim system with 45 mT/m gradients and 200 T/m/s slew rates (Siemens, Erlangen, Germany). All images were obtained parallel to the anterior-posterior commissure plane with no interslice gap. DTI images were acquired with a twice-refocused spin echo single-shot Echo Planar Imaging sequence (Reese et al., 2003) to minimize eddy current-induced image distortions. The protocol consisted of a set of 30 non-collinear diffusion-weighted acquisitions with *b*-value = 1,000 s/mm^2^ and two T2-weighted *b*-value = 0 s/mm^2^ acquisitions, repeated two times (TR/TE = 5,500/98 ms, 128 × 128 matrix, 1.7 × 1.7 mm^2^ in-plane resolution, FA = 90, GRAPPA acceleration factor 2, and bandwidth of 1698 Hz/Px, comprising 40 3-mm-thick slices).

### DTI analysis

DTI allows inferences about WM microstructure *in vivo* by quantifying the magnitude and directionality of diffusion of water within a tissue (Beaulieu, 2002). Visual checks were performed on every volume of the raw data of every participant by AZB and TC. In case a diffusion scan contained more than two volumes with artifacts, these volumes as well as the corresponding *b*-vectors and *b*-values were removed before processing. If artifacts were found in more than two volumes, such datasets were excluded from analyses, resulting in 174 good quality pre-post datasets (Supplementary Material 1).

Next, DTI data were processed using the FSL Diffusion Toolbox v.3.0 (FDT: http://www.fmrib.ox.ac.uk/fsl) in a standard multistep procedure, including: (a) motion and eddy current correction of the images and corresponding b-vectors, (b) removal of the skull and non-brain tissue using the Brain Extraction Tool (Smith, 2002), and (c) voxel-by-voxel calculation of the diffusion tensors. Using the diffusion tensor information, FA maps were computed using DTIFit within the FDT. All motion- and eddy-current outputs, as well as FA images were visually inspected.

We used tract-based spatial statistics (TBSS, a toolbox within FSL v5.0.1), to create a representation of main WM tracts common to all subjects (also commonly known as the WM “skeleton”) (Tract-Based Spatial Statistics, Smith et al., 2004, 2006, 2007). This included: (1) nonlinear alignment of each participant's FA volume to the 1 × 1 × 1 mm^3^ standard Montreal Neurological Institute (MNI152) space via the FMRIB58_FA template using the FMRIB's Nonlinear Registration Tool (FNIRT, Rueckert et al., 1999), (2) calculation of the mean of all aligned FA images, (3) creation of the WM “skeleton” by perpendicular non-maximum-suppression of the mean FA image and setting the FA threshold to 0.25, and (4) perpendicular projection of the highest FA value (local center of the tract) onto the skeleton, separately for each subject. The same procedures were applied to baseline and post-intervention images.

Next, we selected regions of interest on the TBSS skeleton with the use of the DTI WM atlas to probe FA in the core parts of the selected tracts (Burzynska et al., 2013). The 20 WM tracts and their respective acronyms are specified in **Figure 3**. The prefrontal WM region was defined as *y* > 12 in MNI coordinate space and whole WM included the whole TBSS skeleton. Corpus callosum was segmented as in Hofer and Frahm (2006).

### Virginia cognitive aging battery

We administered a cognitive battery as described in the Virginia Cognitive Aging Project to measure latent constructs of fluid intelligence (Raven's Advanced Progressive Matrices test), perceptual speed (letter comparison, patter comparison, digit symbol substitution), and vocabulary (vocabulary, picture vocabulary, synonym vocabulary, and antonym vocabulary; Salthouse and Ferrer-Caja, 2003; Salthouse, 2004, 2005, 2010; Supplementary Material 2). The computer-based tasks were programmed in E-Prime version 1.1 (Psychology Software Tools, Pittsburgh, PA) and administered on computers with 17” cathode ray tube monitors. Several participants had missing or invalid data for single tasks 0.165 out of 174 had complete cognitive data.

To obtain components representing the four cognitive constructs and to confirm the validity of task structure as presented in Salthouse and Ferrer-Caja (2003), we performed principal component analysis (PCA) with varimax rotation. Individual scores on each of the 16 tasks were first screened for outliers and winsorized [maximum 5 cases out of 174 (<3%) were adjusted per variable]. The resulting constructs are presented in Supplementary Material 2. The component scores were saved as variables.

### Cardiorespiratory fitness (CRF)

Baseline CRF was used to predict the change in FA over 6-months of intervention. Participants received consent from their personal physician before cardiorespiratory fitness testing was conducted. CRF (VO_2_ peak) was assessed by graded maximal exercise testing on a motor-driven treadmill. The protocol involves walking at a self-selected pace with incremental grades of 2–3% every 2 min. Measurements of oxygen uptake, heart rate and blood pressure were constantly monitored. Oxygen uptake (VO_2_) was measured from expired air samples taken at 30-s intervals until a peak or maximum VO_2_ (VO_2_ peak or max) was attained; test termination was determined by symptom limitation, volitional exhaustion, and/or attainment of VO_2_ peak as per ACSM guidelines (acsm.org). Due to technical problems CRF data was not collected from 2 participants, resulting in *n* = 172 for CRF.

### Objective physical activity (PA) assessment

Quantitative baseline PA was used to assess baseline lifestyle PA and predict the change in FA over 6-months of intervention. PA was measured by accelerometer (Model GT1M or GT3X; Actigraph, Pensacola, FL). Each participant was instructed to wear the accelerometer on the non-dominant hip during waking hours for 7 consecutive days, and record the time that they wore the device each day on a log. When scored with an interruption period of 60 min, those with at least 10 h of wear time on at least 3 days were retained in analyses (Troiano et al., 2008; Peterson et al., 2010). These data were downloaded as activity counts, which represent raw accelerations that have been summed over a specific epoch length (e.g., 60 s), and these counts vary based on frequency and intensity of the recorded acceleration (Fanning et al., 2016). Next, these data were processed using cut points designed specifically for older adults (Copeland and Esliger, 2009) such that 50 or fewer counts per minute corresponded with sedentary behavior, 51–1,040 counts per minute corresponded to light PA, and 1,041 counts or greater represented moderate-to-vigorous PA (MVPA), related to increased heart rate and ventilation (Rejeski et al., 2016). Five participants did not have valid accelerometer data, resulting in final sample of 169 for PA.

### Statistical analyses

To investigate the effects of time and time x group interactions for the group of 174 participants we used repeated measures ANOVA in SPSS v.24 as our data were complete and the interval between baseline and post-intervention measurements was same for all participants (Liu et al., 2012).

## Results

### Sample characteristics at baseline

First, we tested whether randomization based on age and gender resulted in same baseline characteristics among the four intervention groups. Table 1 shows the demographics for the final sample of 174 people who had good quality of pre and post-intervention diffusion scans. One-way ANOVA revealed that the different intervention groups did not differ at baseline with respect to age, gender, education, BMI, VO_2_ peak (*n* = 172), PA (*N* = 169), and cognitive status (MMSE).

**Table 1:** **Sample characteristics at baseline**.

	**Total**	**Dance**	**Walking**	**Walking + nutrition**	**Active control**	Group difference^*^(*p*-value)
*N*	174	49	40	42	43	–
Age	65.4 ± 4.46	65.88 ± 4.70	64.98 ± 4.00	64.95 ± 4.18	66.72 ± 4.65	0.272
Gender	120 F (69%)	37 F (76%)	27 F (68%)	27 F (64%)	29 F (67%)	0.688
Education (years)	15.92 ± 3.00	3.86 ± 1.26	3.90 ± 1.08	3.90 ± 1.19	4.12 ± 1.10	0.474
MMSE	28.52 ± 1.46	28.43 ± 1.59	28.6 ± 1.55	28.50 ± 1.33	28.56 ± 1.39	0.952
VO_2_ peak [ml/min/kg]	19.77 ± 4.29	20.14 ± 4.34	20.67 ± 4.83	20.28 ± 4.31	19.76 ± 4.45	0.801
***PA***						
Light PA [hours daily]	4.53 ± 1.10	4.48 ± 1.09	4.50 ± 1.05	4.61 ± 1.34	4.56 ± 0.93	0.946
Sedentary [hours daily]	8.96 ± 1.41	9.24 ± 70.84	8.80 ± 1.64	8.88 ± 1.28	8.88 ± 1.57	0.480
MVPA [minutes daily]	45.14 ± 28.42	42.15 ± 23.02	51.73 ± 34.26	42.98 ± 29.12	44.84 ± 27.64	0.434
BMI [kg/m^2^]	30.57 ± 5.49	30.58 ± 5.94	31.18 ± 4.93	29.81 ± 4.85	30.34 ± 6.04	0.720

All results: M ± SD; F, female;

* *Result of one-way ANOVA with group as fixed factor; MMSE, Mini-mental State Examination; PA, physical activity; MVPA, moderate to vigorous PA*.

Next, we investigated baseline characteristics in various diffusion measures. Regional values for FA, RD, MD, and AD for the whole sample and for each of the intervention groups are shown in the Supplementary Material 3. Using one-way ANOVA, we determined that FA, RD, AD and MD measures did not significantly differ among the four intervention groups at baseline in any of the region (for details see Supplementary Tables 3.1 to 3.4).

### Changes in WM diffusivity characteristics over 6-months

Next, we examined change in FA, RD, AD, and MD over 6-months of the intervention. We found that time had a significant effect on all diffusivity values in multiple regions. Table 2 represents mean regional % change for the entire sample (*n* = 174, *p* < 0.05, uncorrected) and the Active Control group (no expected change in CRF or MVPA that would affect cognition or brain health). As we observed only one time x group interaction (fornix, see Section Change in diffusivity parameters: the effects of intervention), we consider the results of entire sample consistent with the Active Control.

**Table 2:** **Percentage Δ in FA, RD, AD, and MD during 6-months for the entire sample and the active group**.

**WM region**	***N***	**FA %Δ ± SD**	**RD %Δ ± SD**	**AD %Δ ± SD**	**MD %Δ ± SD**
ACC	174	−**0.69** ± **3.72**	**0.71** ± **3.57**	0.12±3.61	0.39±3.01
	43	−**01.40** ± **3.73**	**1.19** ± **3.49**	0.11±3.39	0.62±2.83
ALIC	174	−**0.53** ± **2.78**	**1.17** ± **4.05**	**0.46** ± **2.66**	**0.76** ± **2.90**
	43	−**0.91** ± **0.77**	**1.90** ± **4.53**	0.74±2.98	**1.24** ± **3.34**
EC	174	−**1.38** ± **3.49**	**1.43** ± **3.23**	**0.61** ± **1.98**	**1.03** ± **2.47**
	43	−**2.04** ± **3.25**	**1.89** ± **2.96**	**0.68** ± **2.13**	**1.31** ± **2.39**
fMAJ	174	0.25±2.27	−**0.78** ± **3.20**	−**0.38** ± **1.97**	−**0.55** ± **1.74**
	43	0.01±2.34	−0.90±3.80	−**0.66** ± **1.62**	−**0.75** ± **1.93**
fMIN	174	0.34±3.43	0.08±3.39	0.11±2.10	0.09±2.60
	43	−0.47±3.56	0.48±3.57	−0.01±1.93	0.24±2.63
FX	174	−**1.39** ± **8.00**	**2.11** ± **5.39**	**0.98** ± **3.75**	**1.61** ± **4.40**
	43	−**3.57** ± **7.04**	**4.08** ± **5.35**	**1.96** ± **3.38**	**3.12** ± **4.23**
gyrRect	174	−0.04±4.02	0.34±3.69	0.17±2.97	0.25±3.02
	43	−0.24±4.33	−0.59±4.01	−0.89±3.06	−0.74±3.22
HIPP	174	−**0.75** ± **5.53**	**2.05** ± **6.84**	**0.83** ± **4.30**	**1.31** ± **4.18**
	43	−0.41±5.48	2.16±7.74	**1.82** ± **4.71**	**1.91** ± **5.14**
ILF_temp	174	−0.39±4.77	0.50±3.58	0.22±3.27	0.36±3.21
	43	−0.87±4.77	1.02±3.53	0.89±3.19	0.95±3.15
IFOF_ILF_occ	174	−**0.57** ± **3.32**	**0.69** ± **3.56**	−0.14±2.91	0.18±2.20
	43	−**0.96** ± **3.05**	0.79±3.27	−0.44±2.58	0.06±2.12
IFOF_UNC	174	−**0.55** ± **3.68**	**1.11** ± **3.48**	**0.57** ± **2.37**	**0.81** ± **2.42**
	43	−0.91±4.44	**1.31** ± **3.46**	0.60±2.34	**0.92** ± **2.28**
PCC	174	0.16±10.96	1.38±11.09	0.75±8.21	0.93±7.92
	43	0.10±10.09	1.37±11.47	0.88±6.81	1.04±7.72
PLIC	174	−**0.85** ± **2.63**	**3.30** ± **7.01**	**1.50** ± **2.87**	**2.10** ± **4.02**
	43	−**1.33** ± **2.58**	**4.08** ± **7.43**	**1.43** ± **3.01**	**2.32** ± **4.25**
cc1	174	−**0.45** ± **2.34**	**1.79** ± **5.06**	**0.71** ± **2.34**	**1.06** ± **2.60**
	43	−0.51±2.40	**1.77** ± **5.15**	**0.88** ± **2.38**	**1.19** ± **2.60**
cc2	174	−0.38±5.22	**1.72** ± **7.39**	**0.52** ± **2.87**	**0.92** ± **3.15**
	43	−0.56±5.59	1.19±7.58	0.07±3.08	0.46±3.24
cc3	174	0.19±5.35	0.71±7.71	0.44±3.77	0.46±3.55
	43	0.26±5.54	−0.01±7.24	−0.15±4.13	−0.16±3.21
cc4	174	0.52±6.21	0.03±8.73	0.52±3.57	0.28±4.49
	43	−0.28±5.53	0.49±8.71	0.08±3.71	0.25±4.31
cc5	174	0.18±1.76	−0.50±8.30	0.15±2.12	−0.06±2.92
	43	−0.08±1.53	0.65±7.40	0.38±2.37	0.44±3.02
SCR	174	−**0.41** ± **2.25**	**0.88** ± **3.15**	**0.31** ± **1.60**	**0.55** ± **2.09**
	43	−**0.81** ± **2.59**	**1.47** ± **3.62**	0.40±1.81	**0.87** ± **2.42**
SLF	174	−0.26±2.21	**0.66** ± **2.25**	**0.43** ± **1.61**	**0.53** ± **1.62**
	43	−0.26±2.39	**0.91** ± **2.39**	**0.75** ± **1.63**	**0.83** ± **1.65**
UNC_pfc	174	−**1.01** ± **4.22**	**0.51** ± **2.80**	−0.21±2.53	0.17±2.36
	43	−1.06±5.12	0.34±2.33	−0.40±2.42	−0.01±1.88
Prefrontal	174	−**0.33** ± **2.03**	**0.46** ± **1.97**	0.15±1.56	**0.30** ± **1.58**
	43	−**0.71** ± **2.24**	0.57±2.05	0.02±1.40	0.30±1.50
Whole WM	174	−**0.38** ± **1.84**	**0.61** ± **1.86**	**0.27** ± **1.21**	**0.44** ± **1.34**
	43	−**0.68** ± **1.84**	**0.85** ± **1.86**	0.32±1.13	**0.58** ± **1.29**

**Figure 1:**
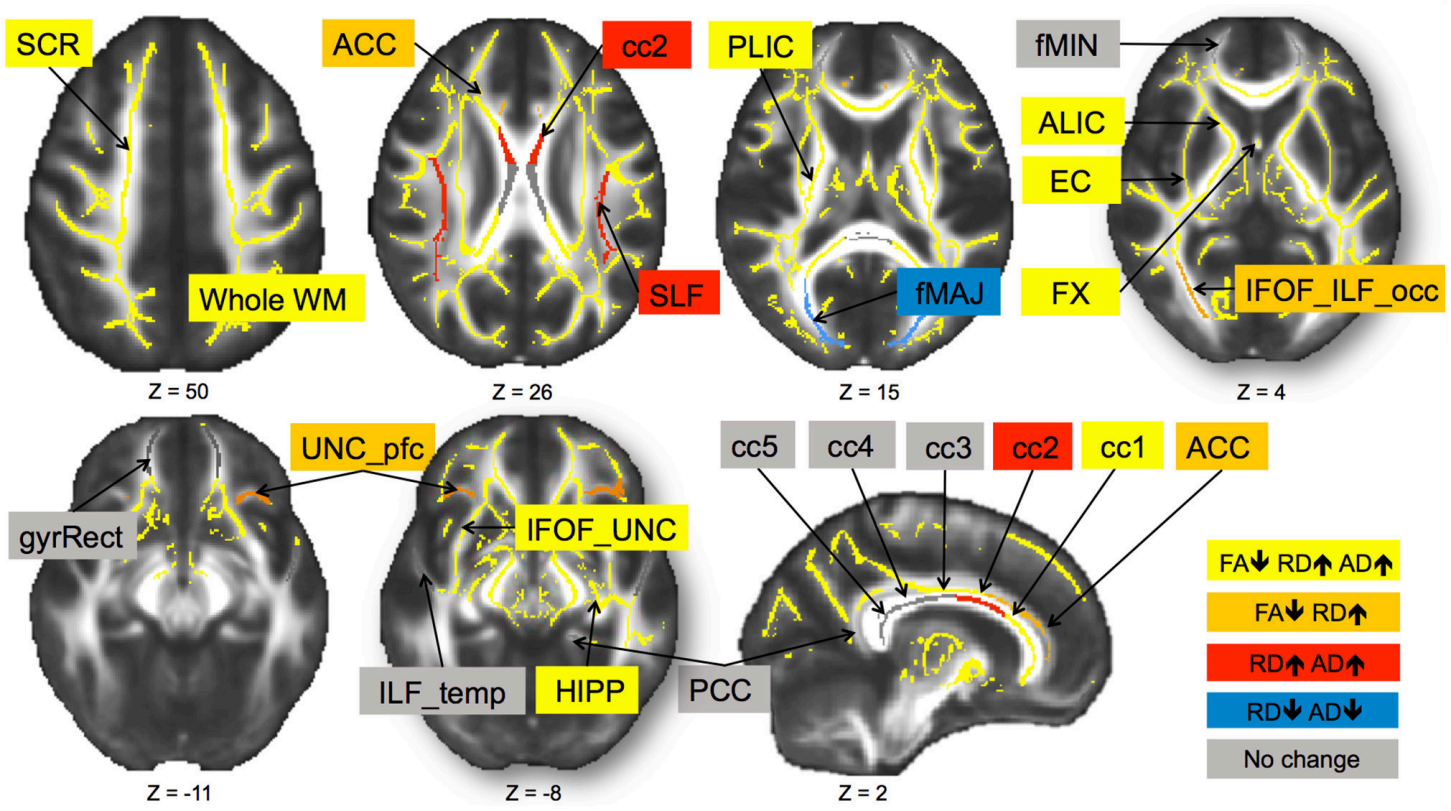
**Change in diffusivity measures over 6-months in healthy older adults 60–79 years old**. Different patterns of overlap of change in diffusivity parameters are represented by different colors. Superior corona radiata (SCR), superior longitudinal fasciculus (SLF), anterior and posterior limb of the internal capsule (ALIC, PLIC), external capsule (EC), fornix (FX), five regions of the corpus callosum (cc1–5), forceps major (fMAJ), forceps minor (fMIN), anterior and posterior cingulum (ACC, PCC), WM containing occipital portion of inferior longitudinal fasciculi and inferior frontal-occipital fasciculi (IFOF_ILF_occ), WM of the straight gyrus (gyrRect), parahippocampal WM (HIPP), ventral prefrontal part of uncinate fasciculus (UNC_pfc), WM containing uncinate and the inferior frontal-occipital fasciculi (IFOF_UNC), and WM of the temporal pole related to inferior longitudinal fasciculus (ILF_temp). Regions are overlaid on the FMRIB58_FA template.

*Regions are listed in Figure 1. Significant effects of time (p < 0.05, uncorrected) are marked in bold*.

In sum, out of 21 regions, FA consistently decreased in 11 regions as well as in the prefrontal WM and the entire skeleton. RD increased in 13 regions as well as in the prefrontal WM and the entire skeleton. AD increased in 10 regions as well as in whole WM. MD increased in 10 regions, as well as in the prefrontal WM and the entire skeleton. This regionally specific pattern of overlap of changes in FA, RD, and AD is summarized in Figure 1 (MD is not included as highly redundant to RD and AD). Only in fMAJ RD, AD, and MD decreased.

### Change in diffusivity parameters: the effects of intervention

**Figure 2:**
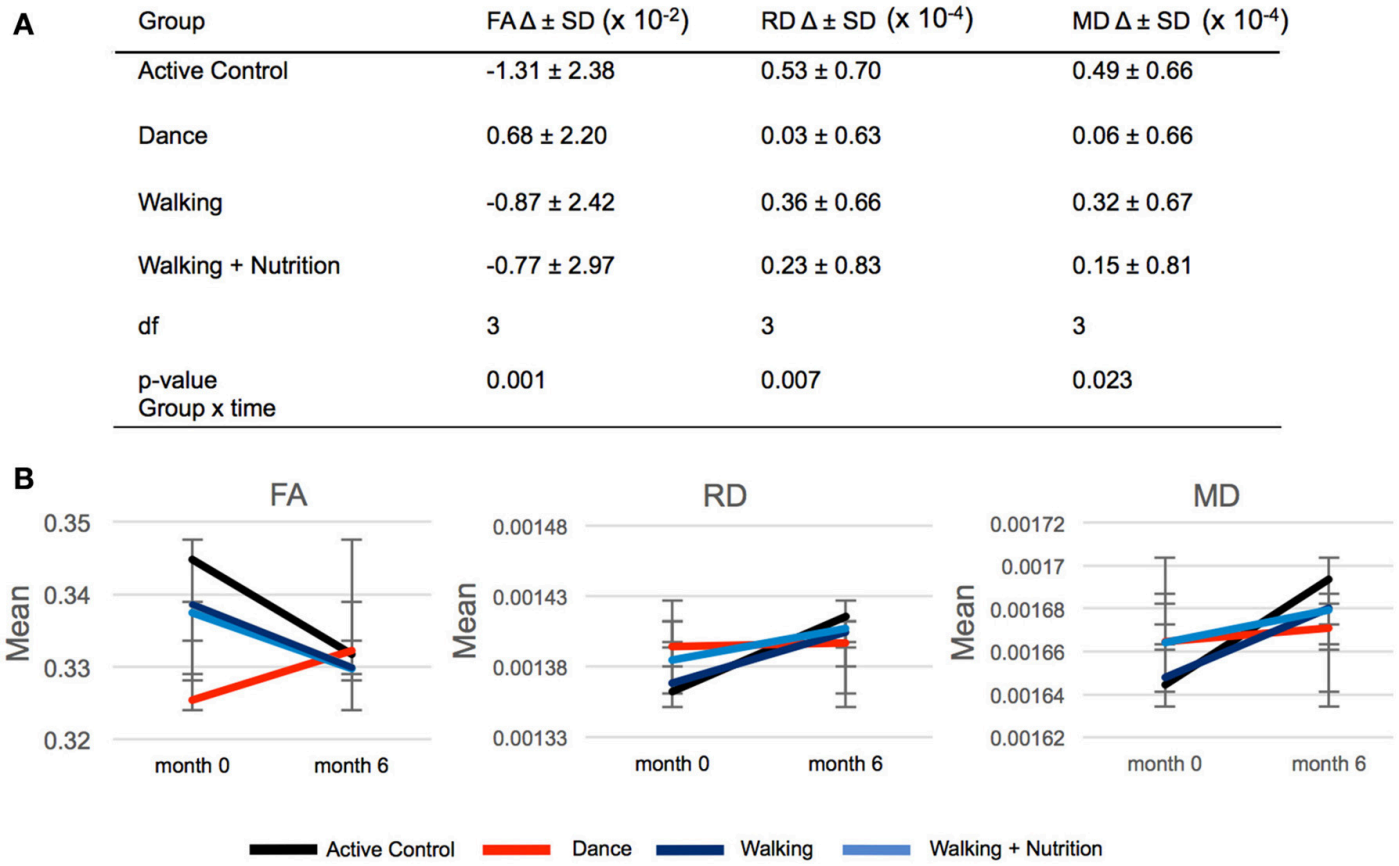
**(A)** Time × group interaction in diffusivity parameters in the fornix in the four intervention groups. SD, standard deviation; Df, degrees of freedom. **(B)** Graphic representation of changes (delta) in FA, RD, and MD during 6-months presented in **(A)**. Error bars: standard deviation.

We used repeated measures ANOVA with time as within-subject factor and the four intervention groups as between-subject factors to investigate differences in FA change between the three intervention groups and the Active Control group (Supplementary Material 4). Out of 21 regions, only the fornix showed significant time x group interaction: *F*_(3, 170)_ = 5.6, *p* = 0.001 (significant after Bonferroni correction of the *p*-value from 0.05 to 0.002). *Post-hoc* pairwise comparisons showed that FA in the fornix decreased in both Walking and the Active Control group, but in the Dance group increased on average by 0.68 × 10^−2^ (Figure 2). We found that this time x group interaction in the fornix was driven by RD and MD: There was a significant effect for RD [*F*_(3, 170)_ = 4.122, *p* = 0.007] and MD [*F*_(3, 170)_ = 3.250, *p* = 0.023], where RD and MD increased to a significantly lesser extent in the Dance group compared to all other (Figure 2). The result on RD and MD was significant after Bonferroni correction of the *p*-value from 0.05 to 0.016. Pairwise *post-hoc* analyses are presented in Supplementary Material 5.

### Relation of change in diffusivity to cognitive performance

**Figure 3:**
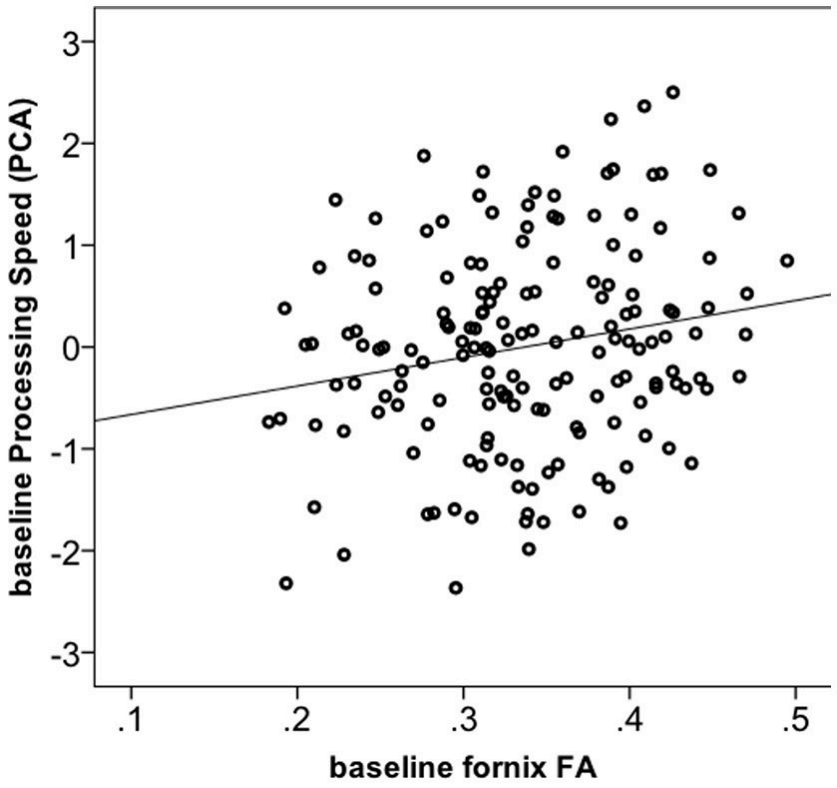
**Correlation between fornix FA and processing speed construct at baseline (*n* = 165)**.

We tested whether the increase in FA in the fornix in the Dance group had cognitive relevance. 165 out of 174 participants had complete cognitive data. Using one-way ANOVA, we found no significant group differences for any of the four cognitive constructs at the baseline (Supplementary Material 6). Next, correlation of baseline fornix FA with the four cognitive constructs yielded a positive association only for processing speed (*r* = 0.19, *p* = 0.013, *n* = 165, 2-tailed; significant after Bonferroni correction to *p* = 0.013 but not significant if controlled for age; Figure 3), but not for memory, vocabulary or reasoning (*p* > 0.05). Therefore, further analyses were restricted to the processing speed. First, we did *post-hoc* correlations between baseline fornix FA and the three tasks within the processing speed construct. They were all positively related to fornix FA: digit symbol (*r* = 0.18, *p* = 0.021, *n* = 173), pattern comparison (*r* = 0.17, *p* = 0.029, *n* = 174) and letter comparison (*r* = 0.15, *p* = 0.047, *n* = 174). These *post-hoc* analyses were not corrected for multiple comparisons. Repeated measures ANOVA on these three tasks revealed significant effect of time for the digit symbol [*F*_(1, 166)_ = 3027.728, *p* = 0.000] and pattern comparison [*F*_(1, 166)_ = 19.165, *p* = 0.002]: all groups showed increased performance over the 6-months of the trial (significant at corrected *p* = 0.016). Given lack of time x group interaction for processing speed, we correlated % change in the task performance with % change in fornix FA, but found no significant effects (*r* = −0.05, *p* = 0.492, *n* = 172).

### Effect of age, CRF, and PA on FA decline over 6-months

**Figure 4:**
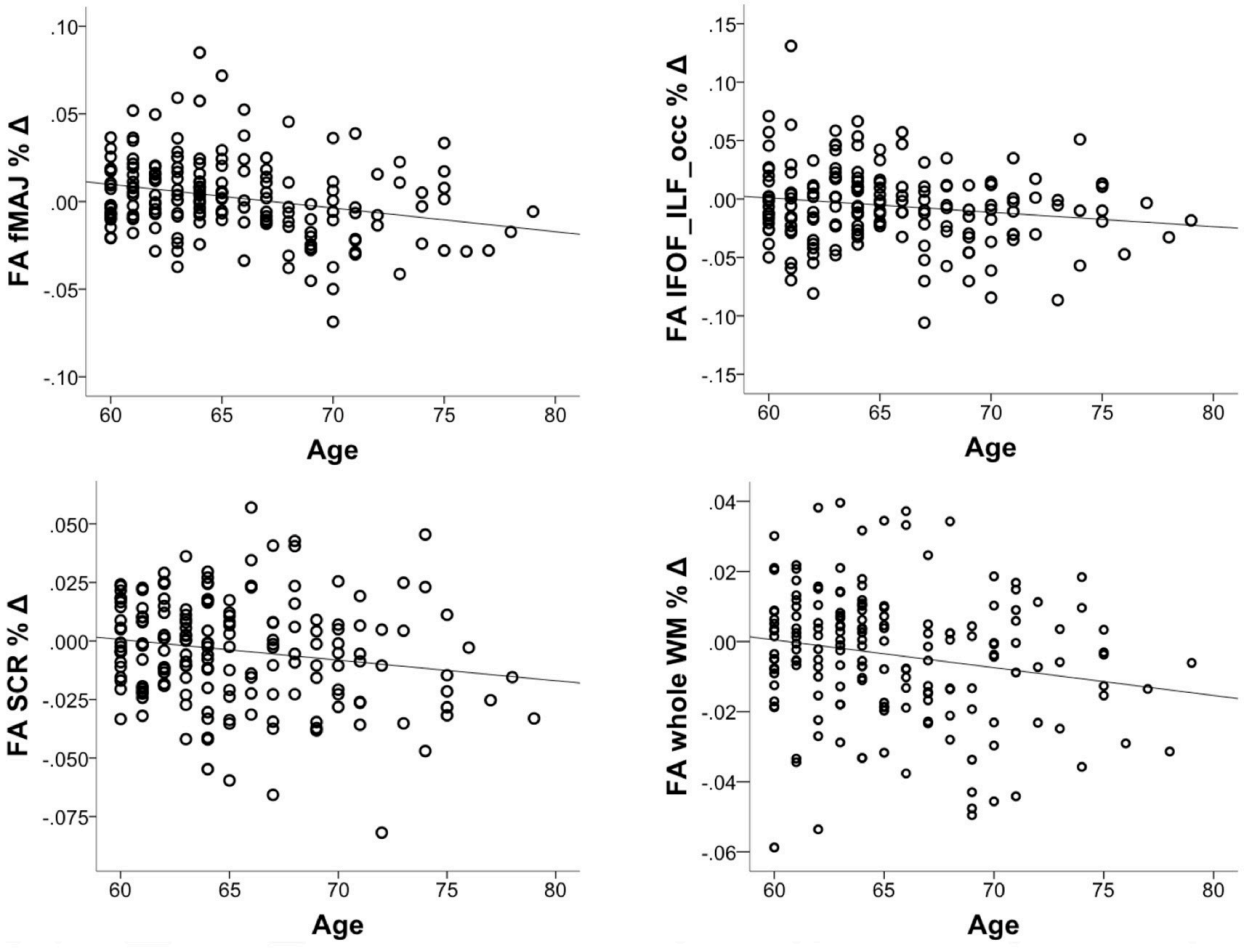
**Correlations between chronological age and % decline in FA over 6-months (*n* = 174)**.

We investigated whether the decrease in FA accelerates in older age. To this aim, we correlated % change in FA with chronological age, controlling for the intervention group. The correlation was significant in fMAJ (*r* = −0.26, *p* = 0.000, *df* = 171), IFOF_ILF_occ (*r* = −0.16, *p* = 0.033, *df* = 171), SCR (*r* = −0.17 *p* = 0.025, *df* = 171), and whole WM (*r* = −0.19, *p* = 013, *df* = 171; Figure 4). Only fMAJ remained significant after Bonferroni correction.

Given the relationships between CRF and PA and WM integrity (Burzynska et al., 2014), we investigated whether greater CRF and levels of PA or sedentary time at baseline were associated with lesser decline in FA over 6-months. A correlation of baseline CRF or light PA and % change in FA, controlled for age, yielded no significant relationships^4^. Greater MVPA was related to more positive change in FA in the prefrontal WM, controlled for age (*r* = 0.15, *p* = 0.048, not corrected). More time spend on sedentary behavior at baseline was associated with more negative change in FA in cc2 section of the corpus callosum (*r* = −0.18, *p* = 0.018, *df* = 166) and in the prefrontal WM (*r* = −0.16, *p* = 0.036, *df* = 166, not corrected), controlled for age. Repeated measures ANOVA showed no significant interaction time × gender interaction, indicating no sex difference in FA decline over 6-months.

## Discussion

We investigated changes in WM microstructure over 6-months in 174 healthy non-demented older adults that underwent four lifestyle interventions. Our main findings are: (1) Only the fornix showed a time x intervention group interaction, namely, FA declined in all groups but increased in the Dance group. Changes in FA were paralleled by changes in RD and MD; (2) FA in the fornix at baseline was related to processing speed, however, there was neither time x intervention group interaction for processing speed nor correlation between change in speed and change in FA; (3) FA decreased over 6-months in the majority of tracts, while RD, AD, and MD increased; (4) There was a spatially-variable pattern of overlap of changes in FA, RD, AD, and MD; (5) Older age was related to greater magnitude of FA decline, especially in fMAJ, IFOF_ILF_occ, SCR, and whole WM; (6) Less sedentariness and more MVPA was associated with less negative change in FA. We discuss these finding in relation to recent longitudinal and intervention studies, theories of neurocognitive aging, and outline future research directions.

### Dance intervention improved FA in the fornix

We found that while FA in the fornix declined over 6-months in most groups, it increased in the Dance group. Greater FA in the fornix at baseline was associated with faster processing speed, also at baseline. We discuss this observation in terms of the role of the fornix in cognition, mechanisms underlying increase in FA, and dance as a complex intervention.

#### Role of the fornix in cognition

The fornix acts as the major output tract of the hippocampus that connects the medial temporal lobes to the mammillary nuclei of the hypothalamus, septal area, and the basal forebrain (Thomas et al., 2011). The fornix is known to play an important role in the encoding, consolidation, and recall of declarative and episodic memory (Thomas et al., 2011). Damage to the fornix as a result of mechanical injury, tumor, or neurodegenerative diseases has been linked to anterograde amnesia and episodic memory impairments (Douet and Chang, 2015). In addition, microstructural and volume changes to the fornix and mammillary bodies have been linked to transition from mild cognitive impairment to clinical Alzheimer's disease (Copenhaver et al., 2006; Mielke et al., 2012; Fletcher et al., 2013; Rémy et al., 2015). In the current study, we observed no relationships between fornix FA and memory. Instead, we found associations with processing speed. We speculate that the relationships between WM microstructure and cognition may be different in clinical samples and in healthy age-related decline. For example, studies including lifespan or healthy older samples showed that fornix integrity correlates not only with episodic memory (Metzler-Baddeley et al., 2011), but also with working memory, motor performance and problem solving (Zahr et al., 2009). As most lifespan studies did not relate fornix integrity to a broader array of cognitive tasks, there is little evidence for the specific role of the fornix in other cognitive domains. Importantly, there is a study that reported specific relationships between fornix integrity and processing speed but not memory in temporal lobe epilepsy patients (Alexander et al., 2014). Together, our results confirm previous associations of WM microstructure and cognitive function in aging and suggest a role of the fornix in cognition beyond long-term memory (Madden et al., 2012).

#### Mechanisms of increased FA

Increases in FA reflect increase in anisotropy of the tissue (Sen and Basser, 2005). This implies changes in the microstructure that would result in more directional diffusion if the water molecules. We showed that this interaction was driven by lack of decrease in RD and the related maintenance of MD. This points to restricted diffusion perpendicular to main fiber direction, which is related to presence and integrity of axonal membrane and myelin. In other words, at the cellular level, the suggested increase in FA is most likely related to stabilization or increase in myelin integrity (Burzynska et al., 2010). There is *in vitro* and animal evidence that repetitive stimulation of certain WM connections, such as in learning of complex motor skills in mice, results in increased myelination (McKenzie et al., 2014). In addition, there is *in vivo* evidence for increases in FA as a result of training in healthy adults (Bengtsson et al., 2005; Scholz et al., 2009; Sampaio-Baptista et al., 2013).

However, there may be an alternative explanation for increased FA, and reduced RD and MD, namely, macrostructural rather than microstructural changes. Fornix is a thin tract surrounded by lateral ventricles and therefore affected by partial volume with cerebrospinal fluid. The Dance intervention might have increased the volume of the fornix, leading to decreased partial volume and decreased RD and MD. Local increases in WM volume and macrostructural integrity have been observed as a result of lifestyle interventions with physical activity (Colcombe et al., 2006; Bolandzadeh et al., 2015). In sum, we speculate that the observed increase in FA results from both macro- and micro-structural reorganization of the fornix.

#### Dance as a complex intervention

Dance is a pleasurable and captivating activity, which involves aerobic exercise, sensorimotor stimulation, and cognitive, visuospatial, social, and emotional engagement. Epidemiological studies found that ballroom dance has also been associated with a protective effective against dementia onset in older adults (Verghese et al., 2003) and reduced depression in community-dwelling older adults with depression (Haboush et al., 2006). Indeed, there is increasing interest in dance as a therapeutic intervention for various clinical groups (Dhami et al., 2015), such as in Parkinson's disease (McNeely et al., 2015) and dementia (Ballesteros et al., 2015; Adam et al., 2016). Our current data indicate that this broad, multimodal stimulation had greater benefit for WM integrity than aerobic exercise alone (i.e., Walking and Walking + Nutrition). This is in line with recent findings that combined exercise and cognitive interventions have more benefit for cognitive, physical, and mental health in older population than each intervention alone (Oswald et al., 2006; Law et al., 2014; Bamidis et al., 2015; Lauenroth et al., 2016). Combined cognitive and physical interventions may also have more long-lasting effects (Rahe et al., 2015). Interestingly, despite positive changes in the WM in the Dance group, we found no cognitive benefit for any of the intervention groups. This may be explained by the observation that changes in neuroimaging outcomes may precede changes in cognition by several years in older population (Jack and Holtzman, 2013). Therefore, improvements in cognition may not be detectable after 6-months of intervention, with most participants improving due to test-retest effect. Future interventions of longer duration are required to test this hypothesis of cognitive benefits following neural changes. Also, available evidence for correlations between decline in FA and cognitive change come from measurements acquired at least 2 years apart (Lövdén et al., 2014; Ritchie et al., 2015; Bender et al., 2016a). Together, although evaluation of decline or increase in WM integrity over 6-months gives insight into short-term neural dynamics in old age, it may not be sufficient to detect robust brain-cognition correlations and effects of intervention groups on cognition.

### WM microstructure changes over 6-months

We observed significant time effects on WM microstructure over a 6-months period in majority of tracts. Currently, this is the shortest period of time over which changes in WM microstructures in healthy older adults have been detected. We discuss our findings in terms of magnitude of annual % change, spatial overlap of changes in FA, RD, AD, and MD, and spatial gradient of change.

#### Magnitude of change

We carefully compared the observed magnitudes of % change over 6-months with annual % changes reported in three previous longitudinal studies and one intervention. For this comparison we chose to focus on changes in diffusivity values for the whole WM as most comparable to global WM or averages across tracts reported in other studies.

We observed the greatest semi-annual change in RD (+0.61, +0.85%), followed by MD (+0.44, +0.58%), FA (–0.38, –0.68%) and AD (+0.27, +0.32%; for whole sample and the active control, respectively). These values are of the same order and a very similar magnitude as annual % change in 203 neurologically healthy (MMSE>25) adults over the span of 3.6 years (Sexton et al., 2014). Specifically, using same tools as in the current study (TBSS) to create representation of major WM tracts, the authors reported the following annual % change for the whole WM skeleton: RD +0.50%, MD +0.30%, FA −0.30%, and AD +0.20% (Sexton et al., 2014). A subsequent tract-based analysis of a subset of this dataset (*n* = 118) yielded the average annual changes of RD +0.60%, MD +0.43%, AD +0.29%, and FA −0.27% (Storsve et al., 2016). Similarly, in a sample of 108 cognitively normal (MMSE>26) adults aged 66–87 measured on average 2.6 years apart, mean annual change in FA over several tracts was −0.5% (Rieckmann et al., 2016). Finally, Voss et al. (2013b) compared changes in lobar diffusivity properties of 55 to 80 years old healthy adults (mMMSE>51) randomized into two intervention groups: aerobic walking group and stretching-tonic active control. We averaged diffusivity values of the 4 lobes to obtain annual % change for the whole WM (TBSS skeleton). The values were +0.43% for RD, −0.42% for FA, and +0.15% for AD for 35 adults in the stretching-toning group, consistent with the above reports.

Together, magnitudes of % annual change are consistent across the existing studies and exceed estimates from cross-sectional designs (Barrick et al., 2010; Lövdén et al., 2014; Rieckmann et al., 2016). The small deviations in magnitude of change seem to depend on analysis method of the diffusion data: skeleton-based used by Sexton et al. (2016), Voss et al. (2013b), and current study, tract-based used by Storsve et al. (2016) and Rieckmann et al. (2016). In addition, sample's age may influence the observed FA decline. Studies reporting greater magnitude of change (Voss et al., 2013b; Rieckmann et al., 2016) included older sample (55+) while Sexton et al. (2014) and Storsve et al. (2016) used adult lifespan sample (age 23–87) to estimate annual change. Thus, greater magnitude of change observed in our study may be due to acceleration of changes in WM diffusivity in the 5th decade and thereafter (Sexton et al., 2014). It remains to be determined whether studying change in center of tracts with maximal tract coherence is more or less sensitive to detecting change in WM microstructure as compared to studying the entire volume of the tracts.

#### Patterns of overlap in FA, RD, AD, and MD changes

We observed a pattern of spatial overlap of changes in different diffusivity parameters. The whole WM (i.e., the skeleton) showed significant decreases in FA overlapping with RD and AD increases (and the resulting increases in MD). This pattern was also present in projection fibers (both limbs of the internal capsule, superior corona radiate), limbic system structures (WM near hippocampus, fornix), association fibers (inferior fronto-occipital fasciculus), and in the commissural fibers (genu corpus callosum). In our earlier cross-sectional work we referred to this pattern of diffusivity changes as “chronic WM degeneration” (Burzynska et al., 2010), as it has been observed in chronic or advanced stages of WM degeneration. This involves an increase in extracellular volume fraction resulting from losses in both axons and myelin (Thomalla et al., 2004; Cosottini et al., 2005; Sen and Basser, 2005; Concha et al., 2006; Lindquist et al., 2007; Sidaros et al., 2008; Sun et al., 2008). In line with our earlier cross-sectional work (Burzynska et al., 2010), we localized this “chronic” pattern of microstructural changes to the genu corpus callosum and association fibers. These structures are known to be most vulnerable to environmental and metabolic challenges due to thin myelin and low oligodendrocyte-to-axon ratio (Pfefferbaum and Sullivan, 2001; Bartzokis, 2004; Bartzokis et al., 2004). While in the cross-sectional study this “chronic” pattern accounted for 24% of WM volume showing age differences in FA (Burzynska et al., 2010), in the current longitudinal analyses the “chronic” pattern dominated the WM skeleton.

We observed decreases in FA in parallel with increases in RD in association fibers (anterior cingulum, occipital part of the inferior fronto-occipital fasciculus, and prefrontal part of the uncinate fasciculus). This pattern of increased diffusivity change perpendicular to the main fiber direction has been associated with myelin loss or degeneration (Song et al., 2002, 2005; Ciccarelli et al., 2006; Burzynska et al., 2010) suggesting that these tracts undergo predominantly short–term myelin changes.

We reported decrease increases in RD and AD, and MD without a net change in FA in the premotor section of the corpus callosum (cc2) and in the superior longitudinal fasciculus. We interpret this pattern as subtle changes in both axons and myelin, which are likely to progress to the “chronic” stage with significant FA decrease. We consider fiber reorganization less likely given increase in MD, indicating decrease in cellular barriers within these WM regions. In one region (fMAJ) we found decreases in both RD and AD. Decrease of diffusivity has been related to increase in tissue density such as in gliosis (Burzynska et al., 2010), or with increased partial volume with surrounding gray matter.

Finally, some regions showed no significant change in diffusivity during 6-months. These include forceps minor (anterior thalamic radiations), WM of the gyrus rectus (fibers of frontal pole and orbitofrontal cortex), inferior longitudinal fasciculus (temporal lobe), posterior cingulum, and the body and splenium of the corpus callosum (motor, sensory and parieto-occipital sections; cc3–cc5). These structures showed only weak age differences in FA or no age difference in our earlier cross-sectional work (Burzynska et al., 2010). Therefore, microstructure in these regions may be relatively stable throughout the adulthood, with subtle decline from early to late adulthood but no significant short-term changes during 7th and 8th decade of life.

In sum, longitudinal patterns of diffusivity changes confirm previous cross-sectional evidence for spatially variable increases in RD, AD, MD, and decreases in FA. In contrast to cross-sectional analyses, 6-months longitudinal data provided no evidence for decreases in MD, RD, and AD (except for fMAJ). Qualitative differences in diffusivity patterns across WM regions between cross-sectional and longitudinal samples suggest region-specific and non-linear time courses of adult age changes in WM microstructure.

#### Spatial gradients of change

There have been several attempts to organize the age-related changes using developmental or anatomical frameworks. Sexton et al. (2014) described an inferior-to superior gradient of lesser-to-greater age related changes. This framework builds on cross-sectional evidence that superior fibers may be more vulnerable to age-related changes (Sullivan et al., 2010a; Sullivan and Pfefferbaum, 2010b) and that WM maturation proceeds from inferior to superior regions (Colby et al., 2011). The related “last in, first out” developmental framework posits that tracts that myelinate last in ontogenic development are most vulnerable and first undergo age-related deterioration (Bartzokis et al., 2010). Prefrontal regions and related association fibers myelinate late as compared to motor and sensory regions. Based on this, the “last in, first out” framework can be refined to anterior-to-posterior gradient of greater to lesser decline (Raz et al., 2005). Clearly, within the corpus callosum, our findings support the anterior-to-posterior, “last in, first out” hypothesis, with the “chronic” pattern of diffusivity changes in the genu and no changes observed in the posterior to middle sections. A similar gradient can be seen within the cingulum bundle. However, the mixed pattern of changes in the remaining tracts does not equivocally support any other developmental or anatomical framework. Namely, over 6-months, we observed changes in both superior, inferior, as well as both anterior and posterior association and projection tracts.

Together, the reported WM changes over a 6-months period will have important implications for planning the timeline of future interventions and longitudinal studies, as well for diagnostic follow-ups. For example, knowing that change is robustly detectable over 6-months, the magnitude of decline in FA or other parameters could be used in identifying accelerated slopes and, therefore, individuals at risk of cognitive decline or conversion to MCI at early stages.

### Effects of age, CRF, and PA on FA decline

We found that FA decline increased in magnitude with advancing age, especially in fMAJ, IFOF_ILF_occ, SCR, and whole WM. This is consistent with previous reports of accelerated WM decline after 5th decade of life (Storsve et al., 2016). We found no effects of gender and CRF on FA changes. Interestingly, we found that adults engaging in more MVPA and spending less time on sedentary behaviors showed less negative change in FA, especially in the prefrontal WM. Prefrontal regions have been shown previously to benefit from exercise interventions (Colcombe et al., 2006; Voss et al., 2013b). There are a number of not mutually exclusive mechanisms of action of active lifestyle on the brain: increased levels of neurotrophic and insulin-like growth factors (Carro et al., 2000; Zoladz et al., 2008; Rasmussen et al., 2009; Voss et al., 2013a), better brain perfusion and cerebrovascular health (Black et al., 1990; Bullitt et al., 2009; Thomas and Baker, 2013), as well as lesser metabolic distress related to reduced sedentary behaviors (Yanagibori et al., 1998; Hamilton et al., 2004; Demiot et al., 2007; Hamburg et al., 2007). Our longitudinal results suggest, similar to our previous cross-sectional findings (Burzynska et al., 2014), that only exercise and avoiding sedentariness can slow down WM age-related WM decline.

We acknowledge that there may be other factors not addressed in the current study, such as genetic polymorphisms or diet that influence the magnitude of WM decline in aging. For example, greater amyloid burden was found to explain faster FA decline in parahippocampal cingulum, body corpus callosum, and forceps minor in healthy older adults (Rieckmann et al., 2016), and those carrying the APOE-ε4 risk allele may also preferentially benefit from PA (Etnier et al., 2007; Smith et al., 2013). Future randomized clinical trials on larger samples are needed to understand individual differences in WM deterioration in healthy aging.

## Conclusions

In conclusion, we provided first evidence for a dance intervention resulting in increased FA. We attribute this to the fact that dance is a combined cognitive, physical and social training, known to boost intervention outcomes. We found no relation of fornix FA to memory, but a rather unexpected relation to processing speed, suggesting a role of the fornix beyond the memory systems in healthy individuals. Importantly, knowing fornix FA can be increased with dance training may lead to new avenues for early treatments, for example, in genetically inherited dementias, characterized by reduced fornix FA at preclinical, asymptomatic stages (Ringman et al., 2007). We also provided first evidence for robust decline in WM integrity in healthy older adults over only 6-months. Patterns of change in different diffusivity parameters may reflect region-specific histological mechanisms of decline. Our findings support previous reports of accelerated decline with advancing age and for greater susceptibility of anterior than posterior corpus callosum fibers. However, we found no evidence for gender differences or anterior-to-posterior or superior-to-inferior gradient of decline in the whole WM. Importantly, less time spend sitting and more time spent engaging in MVPA was associated with less negative change in FA, providing the first evidence of objectively measured lifestyle activities on change in WM health.

## Author contributions

AB, designed and conducted the study, preprocessed the data, carried out the analyses and wrote the manuscript; YJ, analyzed the data and wrote the manuscript; AMK, collected and preprocessed data; JF, EA, and NG, collected and preprocessed data; TC, preprocessed data; MV assisted in study design, data collection and preprocessing; EM and AFK, designed the study and wrote the manuscript.

## Funding

This work was supported by National Institute on Aging (https://www.nia.nih.gov/; R37 AG025667; AFK) and the Center for Nutrition Learning and Memory at the University of Illinois at Urbana-Champaign (http://cnlm.illinois.edu/; AFK). The funders had no role in study design, data collection and analysis, decision to publish, or preparation of the manuscript.

### Conflict of interest statement

The authors declare that the research was conducted in the absence of any commercial or financial relationships that could be construed as a potential conflict of interest.

## Footnotes

^1^Vik et al. (2015) found decline in fronto-striatal FA over a period of 3 years in 76 adults of 49–80 age at inclusion (only FA for selected tracts was analyzed). Similarly, Pfefferbaum et al. (2014) reported decline in FA across white matter in 56 older individuals over 1–8 years (control sample).

^2^The total sample included 247 participants, but only 174 had good quality pre-post DTI data (see Methods).

^3^The detailed results for time × group effects on cognitive measures will be covered in another publication. Here we use cognitive data only to show cognitive relevance of WM microstructure and its changes over 6-months.

^4^Partial correlations controlling for gender, or age and gender yielded the same results. Correlations between % change in FA and baseline CRF within each gender group were also not significant (gender was included as VO_2_ max or peak differs between genders).
