# Supplementary material for: White Matter Integrity Declined Over 6-Months, but Dance Intervention Improved Integrity of the Fornix of Older Adults
Source: Front Aging Neurosci. 2017 Mar 16;9:59. doi: 10.3389/fnagi.2017.00059 (PMC5352690; doi:10.3389/fnagi.2017.00059)
Supplement: Supplementary file 1 [file DataSheet1.docx]

Supplementary Material

White matter integrity declined over 6-month, but dance intervention improved integrity of the fornix of older adults

**Running title:** Lifestyle interventions and the aging white matter

Agnieszka Z Burzynska*, Yuqin Jiao, Anya Knecht , Jason Fanning, Elizabeth Awick, Tammy Chen, Neha Gothe, Michelle W Voss, Edward McAuley, Arthur F Kramer

*** Correspondence:** Corresponding Author: Agnieszka Z Burzynska, PhD, aga.burzynska@colostate.edu


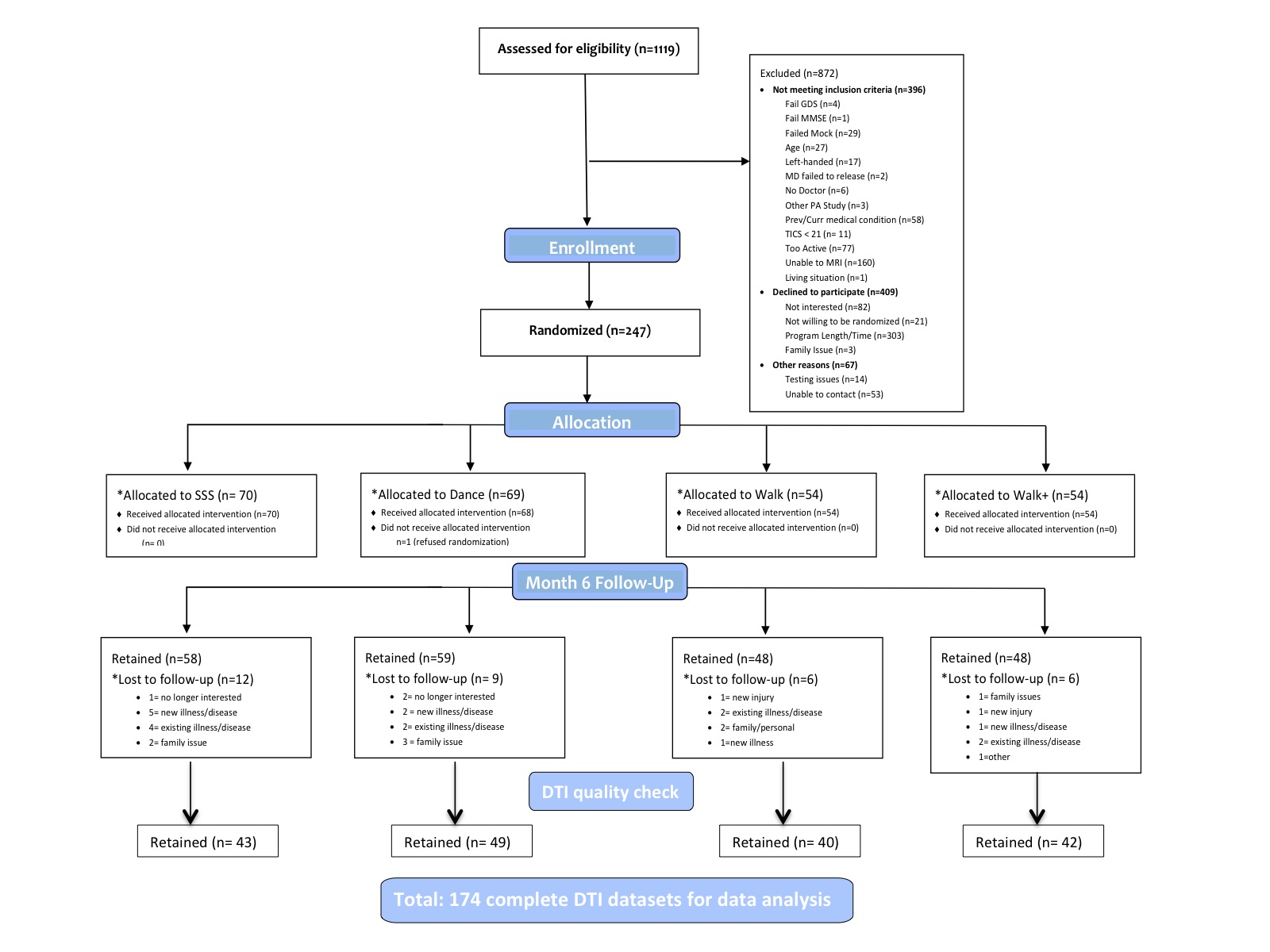
**Supplementary Material 1**

**Figure 1.** Study diagram for the randomization and assessment sessions for four intervention groups.

**Supplementary Material 2**

**PCA on cognitive tasks at the baseline:** In line with previous findings, using principal component analysis (PCA), the 16 cognitive tasks loaded onto four components: vocabulary, fluid reasoning, processing speed, and memory.

**Table 2.** Cognitive battery and the result of dimensionality reduction with PCA for n=164.

| Task | Construct | Description | Administration | Source | Component | | | |
| --- | --- | --- | --- | --- | --- | --- | --- | --- |
|  |  |  |  |  | 1. Fluid abilities | 2. Perceptual Speed | 3. Memory | 4. Vocabulary |
| Matrix reasoning | Fluid intelligence | Select pattern that best completes the missing cell in a matrix | Computer-based | [1] | .682 | – | – | – |
| Shipley abstraction |  | Determine the letters, words, or numbers that best complete a progressive sequence | Paper-pencil | [2] | .533 | – | – | .514 |
| Letter sets |  | Identify which of five groups of letters is different from the others | Computer-based | [3] | .544 | – | – | – |
| Spatial relations | Spatial reasoning | Determine which three dimensional object could be constructed by folding the two dimensional object | Computer-based | [4] | .862 | – | – | – |
| Paper folding |  | Determine the pattern of holes that would result from a sequence of folds and a punch through folded paper | Computer-based | [3] | .768 | – | – | – |
| Form boards |  | Determine shapes needed to fill in a space | Computer-based | [3] | .724 | – | – | – |
| Digit symbol | Perceptual speed | Use a code table to write the correct symbol below each digit | Paper-pencil | [5] | – | .825 | – | – |
| Letter & pattern comparison |  | Same or different comparison of pairs of letter strings/patterns | Paper-pencil | [6] | –  – | .852  .739 | – | – |
| Logical memory | Episodic memory | Recall as many idea units as possible from three stories | Computer-based/paper-pencil | [5] | – | – | .727 | – |
| Free recall |  | Recall as many words as possible across four word trial lists | Computer-based/ paper-pencil | [5] | – | – | .808 | – |
| Paired associates |  | Recall the second words from word pairs | Computer-based/ paper-pencil | [7] | – | – | .826 | – |
| WAIS vocab. | Vocabulary | Define words out loud | Experimenter/ paper-pencil | [5] | – | – | – | .850 |
| Picture vocab. |  | Name the objects presented | Experimenter/ paper-pencil | [8] | – | – | – | .746 |
| Synonym/ antonym |  | Choose the word most similar/opposite in meaning to the target | Computer-based | [9] | – | – | – | .859  .795 |

Note. Columns 6–9: Standardized component loadings from a 4-factor PCA extraction. For clarity, only loadings above 0.50 are displayed. Rotation method: Varimax with Kaiser normalization. Rotation converged in 6 iterations.

In addition, we collected spatial working memory task and task switching. The results from these tasks will be described elsewhere. Here we briefly describe the procedures for the completeness of methods description:

**Spatial Working Memory Paradigm**

At the start of the task, participants were shown a fixation crosshair for 1 second, followed by the appearance of 2, 3, or 4 dots placed in random locations on the screen. Two and 3-dot trials were displayed for 500 milliseconds, and sets of 4 dots were displayed for 1 second. Then, a 3 second fixation crosshair appeared, followed by the appearance of a red dot, which displayed for 2 seconds. Participants had to indicate whether the red dot displayed was in the same location (match) or a different location (non-match) than one of the previously presented black dots, by pressing a designated key on a computer keyboard. Participants completed a total of 120 trials, 40 trials per set size (2, 3, and 4 black dots), each containing 20 match and 20 non-match trials. Participants completed 12 practice trials (6 match, 6 non-match) prior to task administration to familiarize them with the task. Accuracy rates and reaction times were recorded for the 2, 3, and 4 dot conditions and were averaged separately to create mean accuracy and latency scores, respectively.

**Task switching**

Participants were asked to switch back and forth between two different tasks that used the same numeric stimuli, which appeared in the center of the computer screen. In one condition, participants determined whether the digit presented was greater or less than 5; and in the other condition, participants determined whether the digit presented was odd or even. During each trial a blue or pink background of the stimuli instructed participants as to which decision to make (i.e., greater or less than 5, odd/even). Participants received three blocks of stimuli. The first two blocks were the homogenous conditions, in which only one task was performed, and were counterbalanced across participants. The third block consisted of the task-heterogeneous condition in which participants were required to switch between equiprobable task sets on some trials and repeatedly perform the same task over trials in other cases. That is, in the heterogeneous block the two tasks alternated randomly, with seven consecutive trials as the maximum number that were performed repeatedly for each task. Thus, all trials in the heterogeneous block were categorized into either switch or non-switch conditions. White numeric stimuli were presented on a black background for 200 ms, with a 2000 ms inter-stimulus interval from stimulus offset to onset. Participants completed 50 trials in each of the homogenous conditions and 256 trials in the heterogeneous condition. Global switch cost analyses examined differences in reaction time between homogenous and heterogeneous conditions, whereas local switch cost analyses examined differences in reaction time between switch and non-switch trials during the heterogeneous block condition.

(adapted after Hillman et al., 2006; http://www.sciencedirect.com/science/article/pii/S0167876005002291).

# Supplementary Material 3

**Table 3-1.** Comparison of FA values at baseline between the 4 intervention groups (one-way ANOVA, n=174, p-values are not corrected for multiple comparisons).

| **ANOVA** | | | | | | | |
| --- | --- | --- | --- | --- | --- | --- | --- |
|  | | Sum of Squares | df | Mean Square | F | Sig. |  |
| FA_ACC_1 | Between Groups | .001 | 3 | .000 | .346 | .792 |  |
|  | Within Groups | .210 | 170 | .001 |  |  |  |
|  | Total | .211 | 173 |  |  |  |  |
| FA_ALIC_1 | Between Groups | .000 | 3 | .000 | .131 | .942 |  |
|  | Within Groups | .182 | 170 | .001 |  |  |  |
|  | Total | .182 | 173 |  |  |  |  |
| FA_CC_1 | Between Groups | .001 | 3 | .000 | .236 | .871 |  |
|  | Within Groups | .249 | 170 | .001 |  |  |  |
|  | Total | .250 | 173 |  |  |  |  |
| FA_EC_1 | Between Groups | .001 | 3 | .000 | .283 | .838 |  |
|  | Within Groups | .113 | 170 | .001 |  |  |  |
|  | Total | .114 | 173 |  |  |  |  |
| FA_fMAJ_1 | Between Groups | .002 | 3 | .001 | .548 | .650 |  |
|  | Within Groups | .231 | 170 | .001 |  |  |  |
|  | Total | .234 | 173 |  |  |  |  |
| FA_fMIN_1 | Between Groups | .002 | 3 | .001 | .818 | .485 |  |
|  | Within Groups | .126 | 170 | .001 |  |  |  |
|  | Total | .128 | 173 |  |  |  |  |
| FA_FX_1 | Between Groups | .009 | 3 | .003 | .641 | .590 |  |
|  | Within Groups | .810 | 170 | .005 |  |  |  |
|  | Total | .819 | 173 |  |  |  |  |
| FA_gyrRect_1 | Between Groups | .001 | 3 | .000 | .232 | .874 |  |
|  | Within Groups | .131 | 170 | .001 |  |  |  |
|  | Total | .132 | 173 |  |  |  |  |
| FA_HIPP_st_1 | Between Groups | .004 | 3 | .001 | 1.030 | .381 |  |
|  | Within Groups | .196 | 170 | .001 |  |  |  |
|  | Total | .199 | 173 |  |  |  |  |
| FA_ILF_temp_1 | Between Groups | .002 | 3 | .001 | .697 | .555 |  |
|  | Within Groups | .127 | 170 | .001 |  |  |  |
|  | Total | .129 | 173 |  |  |  |  |
| FA_IFOF_ILF_occ_1 | Between Groups | .003 | 3 | .001 | .531 | .662 |  |
|  | Within Groups | .297 | 170 | .002 |  |  |  |
|  | Total | .300 | 173 |  |  |  |  |
| FA_IFOF_UNC_1 | Between Groups | .002 | 3 | .001 | .553 | .647 |  |
|  | Within Groups | .214 | 170 | .001 |  |  |  |
|  | Total | .216 | 173 |  |  |  |  |
| FA_PCC_1 | Between Groups | .003 | 3 | .001 | .285 | .836 |  |
|  | Within Groups | .533 | 170 | .003 |  |  |  |
|  | Total | .536 | 173 |  |  |  |  |
| FA_PLIC_1 | Between Groups | .005 | 3 | .002 | 1.828 | .144 |  |
|  | Within Groups | .140 | 170 | .001 |  |  |  |
|  | Total | .144 | 173 |  |  |  |  |
| FA_prefrontal_1 | Between Groups | .001 | 3 | .000 | .622 | .602 |  |
|  | Within Groups | .086 | 170 | .001 |  |  |  |
|  | Total | .087 | 173 |  |  |  |  |
| FA_reg1cc_1 | Between Groups | .000 | 3 | .000 | .017 | .997 |  |
|  | Within Groups | .250 | 170 | .001 |  |  |  |
|  | Total | .250 | 173 |  |  |  |  |
| FA_reg2cc_1 | Between Groups | .008 | 3 | .003 | .817 | .486 |  |
|  | Within Groups | .547 | 170 | .003 |  |  |  |
|  | Total | .555 | 173 |  |  |  |  |
| FA_reg3cc_1 | Between Groups | .007 | 3 | .002 | .563 | .640 |  |
|  | Within Groups | .728 | 170 | .004 |  |  |  |
|  | Total | .735 | 173 |  |  |  |  |
| FA_reg4cc_1 | Between Groups | .001 | 3 | .000 | .042 | .988 |  |
|  | Within Groups | 1.042 | 170 | .006 |  |  |  |
|  | Total | 1.042 | 173 |  |  |  |  |
| FA_reg5cc_1 | Between Groups | .001 | 3 | .000 | .560 | .642 |  |
|  | Within Groups | .137 | 170 | .001 |  |  |  |
|  | Total | .139 | 173 |  |  |  |  |
| FA_SCR_1 | Between Groups | .002 | 3 | .001 | .592 | .621 |  |
|  | Within Groups | .159 | 170 | .001 |  |  |  |
|  | Total | .161 | 173 |  |  |  |  |
| FA_skel_1 | Between Groups | .001 | 3 | .000 | .683 | .563 |  |
|  | Within Groups | .066 | 170 | .000 |  |  |  |
|  | Total | .067 | 173 |  |  |  |  |
| FA_SLF_1 | Between Groups | .001 | 3 | .000 | .556 | .645 |  |
|  | Within Groups | .115 | 170 | .001 |  |  |  |
|  | Total | .116 | 173 |  |  |  |  |
| FA_UNC_pfc_1 | Between Groups | .001 | 3 | .000 | .371 | .774 |  |
|  | Within Groups | .119 | 170 | .001 |  |  |  |
|  | Total | .119 | 173 |  |  |  |  |
|  |  |  |  |  |  |  |  |

**Table 3-2.** Comparison of RD values at baseline between the 4 intervention groups (one-way ANOVA, n=174, p-values are not corrected for multiple comparisons).

| **ANOVA** | | | | | | | | | | | | | |
| --- | --- | --- | --- | --- | --- | --- | --- | --- | --- | --- | --- | --- | --- |
|  | | | Sum of Squares | | df | | Mean Square | | F | | Sig. | |  |
| RD_ACC_1 | Between Groups | .000 | | 3 | | .000 | | 1.309 | | .273 | |  |  |
|  | Within Groups | .000 | | 170 | | .000 | |  | |  | |  |  |
|  | Total | .000 | | 173 | |  | |  | |  | |  |  |
| RD_ALIC_1 | Between Groups | .000 | | 3 | | .000 | | .960 | | .413 | |  |  |
|  | Within Groups | .000 | | 170 | | .000 | |  | |  | |  |  |
|  | Total | .000 | | 173 | |  | |  | |  | |  |  |
| RD_CC_1 | Between Groups | .000 | | 3 | | .000 | | .255 | | .857 | |  |  |
|  | Within Groups | .000 | | 170 | | .000 | |  | |  | |  |  |
|  | Total | .000 | | 173 | |  | |  | |  | |  |  |
| RD_EC_1 | Between Groups | .000 | | 3 | | .000 | | 1.214 | | .306 | |  |  |
|  | Within Groups | .000 | | 170 | | .000 | |  | |  | |  |  |
|  | Total | .000 | | 173 | |  | |  | |  | |  |  |
| RD_fMAJ_1 | Between Groups | .000 | | 3 | | .000 | | .733 | | .533 | |  |  |
|  | Within Groups | .000 | | 170 | | .000 | |  | |  | |  |  |
|  | Total | .000 | | 173 | |  | |  | |  | |  |  |
| RD_fMIN_1 | Between Groups | .000 | | 3 | | .000 | | 1.077 | | .360 | |  |  |
|  | Within Groups | .000 | | 170 | | .000 | |  | |  | |  |  |
|  | Total | .000 | | 173 | |  | |  | |  | |  |  |
| RD_FX_1 | Between Groups | .000 | | 3 | | .000 | | .091 | | .965 | |  |  |
|  | Within Groups | .000 | | 170 | | .000 | |  | |  | |  |  |
|  | Total | .000 | | 173 | |  | |  | |  | |  |  |
| RD_gyrRect_1 | Between Groups | .000 | | 3 | | .000 | | .870 | | .458 | |  |  |
|  | Within Groups | .000 | | 170 | | .000 | |  | |  | |  |  |
|  | Total | .000 | | 173 | |  | |  | |  | |  |  |
| RD_HIPP_st_1 | Between Groups | .000 | | 3 | | .000 | | .613 | | .607 | |  |  |
|  | Within Groups | .000 | | 170 | | .000 | |  | |  | |  |  |
|  | Total | .000 | | 173 | |  | |  | |  | |  |  |
| RD_ILF_temp_1 | Between Groups | .000 | | 3 | | .000 | | 2.884 | | .037 | |  |  |
|  | Within Groups | .000 | | 170 | | .000 | |  | |  | |  |  |
|  | Total | .000 | | 173 | |  | |  | |  | |  |  |
| RD_IFOF_ILF_occ_1 | Between Groups | .000 | | 3 | | .000 | | .279 | | .841 | |  |  |
|  | Within Groups | .000 | | 170 | | .000 | |  | |  | |  |  |
|  | Total | .000 | | 173 | |  | |  | |  | |  |  |
| RD_IFOF_UNC_1 | Between Groups | .000 | | 3 | | .000 | | 1.183 | | .318 | |  |  |
|  | Within Groups | .000 | | 170 | | .000 | |  | |  | |  |  |
|  | Total | .000 | | 173 | |  | |  | |  | |  |  |
| RD_PCC_1 | Between Groups | .000 | | 3 | | .000 | | .545 | | .652 | |  |  |
|  | Within Groups | .000 | | 170 | | .000 | |  | |  | |  |  |
|  | Total | .000 | | 173 | |  | |  | |  | |  |  |
| RD_PLIC_1 | Between Groups | .000 | | 3 | | .000 | | 2.476 | | .063 | |  |  |
|  | Within Groups | .000 | | 170 | | .000 | |  | |  | |  |  |
|  | Total | .000 | | 173 | |  | |  | |  | |  |  |
| RD_prefrontal_1 | Between Groups | .000 | | 3 | | .000 | | .916 | | .434 | |  |  |
|  | Within Groups | .000 | | 170 | | .000 | |  | |  | |  |  |
|  | Total | .000 | | 173 | |  | |  | |  | |  |  |
| RD_reg1cc_1 | Between Groups | .000 | | 3 | | .000 | | .017 | | .997 | |  |  |
|  | Within Groups | .000 | | 170 | | .000 | |  | |  | |  |  |
|  | Total | .000 | | 173 | |  | |  | |  | |  |  |
| RD_reg2cc_1 | Between Groups | .000 | | 3 | | .000 | | .736 | | .532 | |  |  |
|  | Within Groups | .000 | | 170 | | .000 | |  | |  | |  |  |
|  | Total | .000 | | 173 | |  | |  | |  | |  |  |
| RD_reg3cc_1 | Between Groups | .000 | | 3 | | .000 | | .528 | | .664 | |  |  |
|  | Within Groups | .000 | | 170 | | .000 | |  | |  | |  |  |
|  | Total | .000 | | 173 | |  | |  | |  | |  |  |
| RD_reg4cc_1 | Between Groups | .000 | | 3 | | .000 | | .096 | | .962 | |  |  |
|  | Within Groups | .000 | | 170 | | .000 | |  | |  | |  |  |
|  | Total | .000 | | 173 | |  | |  | |  | |  |  |
| RD_reg5cc_1 | Between Groups | .000 | | 3 | | .000 | | .627 | | .598 | |  |  |
|  | Within Groups | .000 | | 170 | | .000 | |  | |  | |  |  |
|  | Total | .000 | | 173 | |  | |  | |  | |  |  |
| RD_SCR_1 | Between Groups | .000 | | 3 | | .000 | | .899 | | .443 | |  |  |
|  | Within Groups | .000 | | 170 | | .000 | |  | |  | |  |  |
|  | Total | .000 | | 173 | |  | |  | |  | |  |  |
| RD_skel_1 | Between Groups | .000 | | 3 | | .000 | | 1.275 | | .285 | |  |  |
|  | Within Groups | .000 | | 170 | | .000 | |  | |  | |  |  |
|  | Total | .000 | | 173 | |  | |  | |  | |  |  |
| RD_SLF_1 | Between Groups | .000 | | 3 | | .000 | | .768 | | .513 | |  |  |
|  | Within Groups | .000 | | 170 | | .000 | |  | |  | |  |  |
|  | Total | .000 | | 173 | |  | |  | |  | |  |  |
| RD_UNC_pfc_1 | Between Groups | .000 | | 3 | | .000 | | .925 | | .430 | |  |  |
|  | Within Groups | .000 | | 170 | | .000 | |  | |  | |  |  |
|  | Total | .000 | | 173 | |  | |  | |  | |  |  |

**Table 3-3.** Comparison of AD values at baseline between the 4 intervention groups (one-way ANOVA, n=174, p-values are not corrected for multiple comparisons).

| **ANOVA** | | | | | | | |
| --- | --- | --- | --- | --- | --- | --- | --- |
|  | | Sum of Squares | df | Mean Square | F | Sig. |  |
| AD_ACC_1 | Between Groups | .000 | 3 | .000 | 1.900 | .131 |  |
|  | Within Groups | .000 | 170 | .000 |  |  |  |
|  | Total | .000 | 173 |  |  |  |  |
| AD_ALIC_1 | Between Groups | .000 | 3 | .000 | 3.359 | .020 |  |
|  | Within Groups | .000 | 170 | .000 |  |  |  |
|  | Total | .000 | 173 |  |  |  |  |
| AD_CC_1 | Between Groups | .000 | 3 | .000 | .336 | .800 |  |
|  | Within Groups | .000 | 170 | .000 |  |  |  |
|  | Total | .000 | 173 |  |  |  |  |
| AD_EC_1 | Between Groups | .000 | 3 | .000 | 2.562 | .057 |  |
|  | Within Groups | .000 | 170 | .000 |  |  |  |
|  | Total | .000 | 173 |  |  |  |  |
| AD_fMAJ_1 | Between Groups | .000 | 3 | .000 | 1.187 | .316 |  |
|  | Within Groups | .000 | 170 | .000 |  |  |  |
|  | Total | .000 | 173 |  |  |  |  |
| AD_fMIN_1 | Between Groups | .000 | 3 | .000 | .579 | .629 |  |
|  | Within Groups | .000 | 170 | .000 |  |  |  |
|  | Total | .000 | 173 |  |  |  |  |
| AD_FX_1 | Between Groups | .000 | 3 | .000 | .039 | .990 |  |
|  | Within Groups | .000 | 170 | .000 |  |  |  |
|  | Total | .000 | 173 |  |  |  |  |
| AD_gyrRect_1 | Between Groups | .000 | 3 | .000 | 1.304 | .275 |  |
|  | Within Groups | .000 | 170 | .000 |  |  |  |
|  | Total | .000 | 173 |  |  |  |  |
| AD_HIPP_st_1 | Between Groups | .000 | 3 | .000 | 2.177 | .093 |  |
|  | Within Groups | .000 | 170 | .000 |  |  |  |
|  | Total | .000 | 173 |  |  |  |  |
| AD_ILF_temp_1 | Between Groups | .000 | 3 | .000 | 3.530 | .016 |  |
|  | Within Groups | .000 | 170 | .000 |  |  |  |
|  | Total | .000 | 173 |  |  |  |  |
| AD_IFOF_ILF_occ_1 | Between Groups | .000 | 3 | .000 | 1.091 | .354 |  |
|  | Within Groups | .000 | 170 | .000 |  |  |  |
|  | Total | .000 | 173 |  |  |  |  |
| AD_IFOF_UNC_1 | Between Groups | .000 | 3 | .000 | 1.459 | .227 |  |
|  | Within Groups | .000 | 170 | .000 |  |  |  |
|  | Total | .000 | 173 |  |  |  |  |
| AD_PCC_1 | Between Groups | .000 | 3 | .000 | .330 | .804 |  |
|  | Within Groups | .000 | 170 | .000 |  |  |  |
|  | Total | .000 | 173 |  |  |  |  |
| AD_PLIC_1 | Between Groups | .000 | 3 | .000 | 1.802 | .149 |  |
|  | Within Groups | .000 | 170 | .000 |  |  |  |
|  | Total | .000 | 173 |  |  |  |  |
| AD_prefrontal_1 | Between Groups | .000 | 3 | .000 | 1.361 | .256 |  |
|  | Within Groups | .000 | 170 | .000 |  |  |  |
|  | Total | .000 | 173 |  |  |  |  |
| AD_reg1cc_1 | Between Groups | .000 | 3 | .000 | .528 | .664 |  |
|  | Within Groups | .000 | 170 | .000 |  |  |  |
|  | Total | .000 | 173 |  |  |  |  |
| AD_reg2cc_1 | Between Groups | .000 | 3 | .000 | .478 | .698 |  |
|  | Within Groups | .000 | 170 | .000 |  |  |  |
|  | Total | .000 | 173 |  |  |  |  |
| AD_reg3cc_1 | Between Groups | .000 | 3 | .000 | .614 | .607 |  |
|  | Within Groups | .000 | 170 | .000 |  |  |  |
|  | Total | .000 | 173 |  |  |  |  |
| AD_reg4cc_1 | Between Groups | .000 | 3 | .000 | .653 | .582 |  |
|  | Within Groups | .000 | 170 | .000 |  |  |  |
|  | Total | .000 | 173 |  |  |  |  |
| AD_reg5cc_1 | Between Groups | .000 | 3 | .000 | .087 | .967 |  |
|  | Within Groups | .000 | 170 | .000 |  |  |  |
|  | Total | .000 | 173 |  |  |  |  |
| AD_SCR_1 | Between Groups | .000 | 3 | .000 | .566 | .638 |  |
|  | Within Groups | .000 | 170 | .000 |  |  |  |
|  | Total | .000 | 173 |  |  |  |  |
| AD_skel_1 | Between Groups | .000 | 3 | .000 | 2.293 | .080 |  |
|  | Within Groups | .000 | 170 | .000 |  |  |  |
|  | Total | .000 | 173 |  |  |  |  |
| AD_SLF_1 | Between Groups | .000 | 3 | .000 | 1.112 | .346 |  |
|  | Within Groups | .000 | 170 | .000 |  |  |  |
|  | Total | .000 | 173 |  |  |  |  |
| AD_UNC_pfc_1 | Between Groups | .000 | 3 | .000 | 1.138 | .335 |  |
|  | Within Groups | .000 | 170 | .000 |  |  |  |
|  | Total | .000 | 173 |  |  |  |  |

**Table 3-4.** Comparison of MD values at baseline between the 4 intervention groups (one-way ANOVA, n=174, p-values are not corrected for multiple comparisons).

| **ANOVA** | | | | | | | |
| --- | --- | --- | --- | --- | --- | --- | --- |
|  | | Sum of Squares | df | Mean Square | F | Sig. |  |
| MD_ACC_1 | Between Groups | .000 | 3 | .000 | 1.853 | .139 |  |
|  | Within Groups | .000 | 170 | .000 |  |  |  |
|  | Total | .000 | 173 |  |  |  |  |
| MD_ALIC_1 | Between Groups | .000 | 3 | .000 | 2.084 | .104 |  |
|  | Within Groups | .000 | 170 | .000 |  |  |  |
|  | Total | .000 | 173 |  |  |  |  |
| MD_CC_1 | Between Groups | .000 | 3 | .000 | .328 | .805 |  |
|  | Within Groups | .000 | 170 | .000 |  |  |  |
|  | Total | .000 | 173 |  |  |  |  |
| MD_EC_1 | Between Groups | .000 | 3 | .000 | 1.867 | .137 |  |
|  | Within Groups | .000 | 170 | .000 |  |  |  |
|  | Total | .000 | 173 |  |  |  |  |
| MD_fMAJ_1 | Between Groups | .000 | 3 | .000 | 1.575 | .197 |  |
|  | Within Groups | .000 | 170 | .000 |  |  |  |
|  | Total | .000 | 173 |  |  |  |  |
| MD_fMIN_1 | Between Groups | .000 | 3 | .000 | 1.032 | .380 |  |
|  | Within Groups | .000 | 170 | .000 |  |  |  |
|  | Total | .000 | 173 |  |  |  |  |
| MD_FX_1 | Between Groups | .000 | 3 | .000 | .053 | .984 |  |
|  | Within Groups | .000 | 170 | .000 |  |  |  |
|  | Total | .000 | 173 |  |  |  |  |
| MD_gyrRect_1 | Between Groups | .000 | 3 | .000 | 1.232 | .300 |  |
|  | Within Groups | .000 | 170 | .000 |  |  |  |
|  | Total | .000 | 173 |  |  |  |  |
| MD_HIPP_st_1 | Between Groups | .000 | 3 | .000 | 1.398 | .245 |  |
|  | Within Groups | .000 | 170 | .000 |  |  |  |
|  | Total | .000 | 173 |  |  |  |  |
| MD_ILF_temp_1 | Between Groups | .000 | 3 | .000 | 3.768 | .012 |  |
|  | Within Groups | .000 | 170 | .000 |  |  |  |
|  | Total | .000 | 173 |  |  |  |  |
| MD_IFOF_ILF_occ_1 | Between Groups | .000 | 3 | .000 | .514 | .673 |  |
|  | Within Groups | .000 | 170 | .000 |  |  |  |
|  | Total | .000 | 173 |  |  |  |  |
| MD_IFOF_UNC_1 | Between Groups | .000 | 3 | .000 | 1.636 | .183 |  |
|  | Within Groups | .000 | 170 | .000 |  |  |  |
|  | Total | .000 | 173 |  |  |  |  |
| MD_PCC_1 | Between Groups | .000 | 3 | .000 | .574 | .633 |  |
|  | Within Groups | .000 | 170 | .000 |  |  |  |
|  | Total | .000 | 173 |  |  |  |  |
| MD_PLIC_1 | Between Groups | .000 | 3 | .000 | 2.799 | .042 |  |
|  | Within Groups | .000 | 170 | .000 |  |  |  |
|  | Total | .000 | 173 |  |  |  |  |
| MD_prefrontal_1 | Between Groups | .000 | 3 | .000 | 1.129 | .339 |  |
|  | Within Groups | .000 | 170 | .000 |  |  |  |
|  | Total | .000 | 173 |  |  |  |  |
| MD_reg1cc_1 | Between Groups | .000 | 3 | .000 | .125 | .945 |  |
|  | Within Groups | .000 | 170 | .000 |  |  |  |
|  | Total | .000 | 173 |  |  |  |  |
| MD_reg2cc_1 | Between Groups | .000 | 3 | .000 | .686 | .562 |  |
|  | Within Groups | .000 | 170 | .000 |  |  |  |
|  | Total | .000 | 173 |  |  |  |  |
| MD_reg3cc_1 | Between Groups | .000 | 3 | .000 | .571 | .635 |  |
|  | Within Groups | .000 | 170 | .000 |  |  |  |
|  | Total | .000 | 173 |  |  |  |  |
| MD_reg4cc_1 | Between Groups | .000 | 3 | .000 | .244 | .865 |  |
|  | Within Groups | .000 | 170 | .000 |  |  |  |
|  | Total | .000 | 173 |  |  |  |  |
| MD_reg5cc_1 | Between Groups | .000 | 3 | .000 | .488 | .691 |  |
|  | Within Groups | .000 | 170 | .000 |  |  |  |
|  | Total | .000 | 173 |  |  |  |  |
| MD_SCR_1 | Between Groups | .000 | 3 | .000 | 1.259 | .290 |  |
|  | Within Groups | .000 | 170 | .000 |  |  |  |
|  | Total | .000 | 173 |  |  |  |  |
| MD_skel_1 | Between Groups | .000 | 3 | .000 | 1.704 | .168 |  |
|  | Within Groups | .000 | 170 | .000 |  |  |  |
|  | Total | .000 | 173 |  |  |  |  |
| MD_SLF_1 | Between Groups | .000 | 3 | .000 | 1.036 | .378 |  |
|  | Within Groups | .000 | 170 | .000 |  |  |  |
|  | Total | .000 | 173 |  |  |  |  |
| MD_UNC_pfc_1 | Between Groups | .000 | 3 | .000 | 1.130 | .338 |  |
|  | Within Groups | .000 | 170 | .000 |  |  |  |
|  | Total | .000 | 173 |  |  |  |  |

**Supplementary Material 4**

**Table 4.** Repeated measure ANOVA with time as within-subject factor, four intervention groups as between-subject factors

| **Univariate Tests** | | | | | | | |
| --- | --- | --- | --- | --- | --- | --- | --- |
| Source | Measure | | Type III Sum of Squares | df | Mean Square | F | Sig. |
| time | acc | Sphericity Assumed | .001 | 1 | .001 | 7.198 | .008 |
|  |  | Greenhouse-Geisser | .001 | 1.000 | .001 | 7.198 | .008 |
|  |  | Huynh-Feldt | .001 | 1.000 | .001 | 7.198 | .008 |
|  |  | Lower-bound | .001 | 1.000 | .001 | 7.198 | .008 |
|  | alic | Sphericity Assumed | .001 | 1 | .001 | 7.214 | .008 |
|  |  | Greenhouse-Geisser | .001 | 1.000 | .001 | 7.214 | .008 |
|  |  | Huynh-Feldt | .001 | 1.000 | .001 | 7.214 | .008 |
|  |  | Lower-bound | .001 | 1.000 | .001 | 7.214 | .008 |
|  | cc | Sphericity Assumed | .000 | 1 | .000 | 1.037 | .310 |
|  |  | Greenhouse-Geisser | .000 | 1.000 | .000 | 1.037 | .310 |
|  |  | Huynh-Feldt | .000 | 1.000 | .000 | 1.037 | .310 |
|  |  | Lower-bound | .000 | 1.000 | .000 | 1.037 | .310 |
|  | ec | Sphericity Assumed | .003 | 1 | .003 | 29.760 | .000 |
|  |  | Greenhouse-Geisser | .003 | 1.000 | .003 | 29.760 | .000 |
|  |  | Huynh-Feldt | .003 | 1.000 | .003 | 29.760 | .000 |
|  |  | Lower-bound | .003 | 1.000 | .003 | 29.760 | .000 |
|  | fMAJ | Sphericity Assumed | .000 | 1 | .000 | 1.770 | .185 |
|  |  | Greenhouse-Geisser | .000 | 1.000 | .000 | 1.770 | .185 |
|  |  | Huynh-Feldt | .000 | 1.000 | .000 | 1.770 | .185 |
|  |  | Lower-bound | .000 | 1.000 | .000 | 1.770 | .185 |
|  | fMIN | Sphericity Assumed | .000 | 1 | .000 | 1.093 | .297 |
|  |  | Greenhouse-Geisser | .000 | 1.000 | .000 | 1.093 | .297 |
|  |  | Huynh-Feldt | .000 | 1.000 | .000 | 1.093 | .297 |
|  |  | Lower-bound | .000 | 1.000 | .000 | 1.093 | .297 |
|  | FX | Sphericity Assumed | .003 | 1 | .003 | 9.019 | .003 |
|  |  | Greenhouse-Geisser | .003 | 1.000 | .003 | 9.019 | .003 |
|  |  | Huynh-Feldt | .003 | 1.000 | .003 | 9.019 | .003 |
|  |  | Lower-bound | .003 | 1.000 | .003 | 9.019 | .003 |
|  | gyrRect | Sphericity Assumed | 1.132E-5 | 1 | 1.132E-5 | .082 | .775 |
|  |  | Greenhouse-Geisser | 1.132E-5 | 1.000 | 1.132E-5 | .082 | .775 |
|  |  | Huynh-Feldt | 1.132E-5 | 1.000 | 1.132E-5 | .082 | .775 |
|  |  | Lower-bound | 1.132E-5 | 1.000 | 1.132E-5 | .082 | .775 |
|  | HIPP_st | Sphericity Assumed | .002 | 1 | .002 | 4.419 | .037 |
|  |  | Greenhouse-Geisser | .002 | 1.000 | .002 | 4.419 | .037 |
|  |  | Huynh-Feldt | .002 | 1.000 | .002 | 4.419 | .037 |
|  |  | Lower-bound | .002 | 1.000 | .002 | 4.419 | .037 |
|  | ILF_temp | Sphericity Assumed | .000 | 1 | .000 | 1.965 | .163 |
|  |  | Greenhouse-Geisser | .000 | 1.000 | .000 | 1.965 | .163 |
|  |  | Huynh-Feldt | .000 | 1.000 | .000 | 1.965 | .163 |
|  |  | Lower-bound | .000 | 1.000 | .000 | 1.965 | .163 |
|  | IFOF_ILF_occ | Sphericity Assumed | .001 | 1 | .001 | 6.204 | .014 |
|  |  | Greenhouse-Geisser | .001 | 1.000 | .001 | 6.204 | .014 |
|  |  | Huynh-Feldt | .001 | 1.000 | .001 | 6.204 | .014 |
|  |  | Lower-bound | .001 | 1.000 | .001 | 6.204 | .014 |
|  | IFOF_UNC | Sphericity Assumed | .001 | 1 | .001 | 4.630 | .033 |
|  |  | Greenhouse-Geisser | .001 | 1.000 | .001 | 4.630 | .033 |
|  |  | Huynh-Feldt | .001 | 1.000 | .001 | 4.630 | .033 |
|  |  | Lower-bound | .001 | 1.000 | .001 | 4.630 | .033 |
|  | PCC | Sphericity Assumed | .000 | 1 | .000 | .178 | .674 |
|  |  | Greenhouse-Geisser | .000 | 1.000 | .000 | .178 | .674 |
|  |  | Huynh-Feldt | .000 | 1.000 | .000 | .178 | .674 |
|  |  | Lower-bound | .000 | 1.000 | .000 | .178 | .674 |
|  | PLIC | Sphericity Assumed | .003 | 1 | .003 | 19.624 | .000 |
|  |  | Greenhouse-Geisser | .003 | 1.000 | .003 | 19.624 | .000 |
|  |  | Huynh-Feldt | .003 | 1.000 | .003 | 19.624 | .000 |
|  |  | Lower-bound | .003 | 1.000 | .003 | 19.624 | .000 |
|  | prefrontal | Sphericity Assumed | .000 | 1 | .000 | 4.858 | .029 |
|  |  | Greenhouse-Geisser | .000 | 1.000 | .000 | 4.858 | .029 |
|  |  | Huynh-Feldt | .000 | 1.000 | .000 | 4.858 | .029 |
|  |  | Lower-bound | .000 | 1.000 | .000 | 4.858 | .029 |
|  | reg1cc | Sphericity Assumed | .001 | 1 | .001 | 7.195 | .008 |
|  |  | Greenhouse-Geisser | .001 | 1.000 | .001 | 7.195 | .008 |
|  |  | Huynh-Feldt | .001 | 1.000 | .001 | 7.195 | .008 |
|  |  | Lower-bound | .001 | 1.000 | .001 | 7.195 | .008 |
|  | reg2cc | Sphericity Assumed | .001 | 1 | .001 | 1.439 | .232 |
|  |  | Greenhouse-Geisser | .001 | 1.000 | .001 | 1.439 | .232 |
|  |  | Huynh-Feldt | .001 | 1.000 | .001 | 1.439 | .232 |
|  |  | Lower-bound | .001 | 1.000 | .001 | 1.439 | .232 |
|  | reg3cc | Sphericity Assumed | 1.465E-5 | 1 | 1.465E-5 | .027 | .869 |
|  |  | Greenhouse-Geisser | 1.465E-5 | 1.000 | 1.465E-5 | .027 | .869 |
|  |  | Huynh-Feldt | 1.465E-5 | 1.000 | 1.465E-5 | .027 | .869 |
|  |  | Lower-bound | 1.465E-5 | 1.000 | 1.465E-5 | .027 | .869 |
|  | reg4cc | Sphericity Assumed | .001 | 1 | .001 | .900 | .344 |
|  |  | Greenhouse-Geisser | .001 | 1.000 | .001 | .900 | .344 |
|  |  | Huynh-Feldt | .001 | 1.000 | .001 | .900 | .344 |
|  |  | Lower-bound | .001 | 1.000 | .001 | .900 | .344 |
|  | reg5cc | Sphericity Assumed | .000 | 1 | .000 | 1.384 | .241 |
|  |  | Greenhouse-Geisser | .000 | 1.000 | .000 | 1.384 | .241 |
|  |  | Huynh-Feldt | .000 | 1.000 | .000 | 1.384 | .241 |
|  |  | Lower-bound | .000 | 1.000 | .000 | 1.384 | .241 |
|  | SCR | Sphericity Assumed | .001 | 1 | .001 | 6.889 | .009 |
|  |  | Greenhouse-Geisser | .001 | 1.000 | .001 | 6.889 | .009 |
|  |  | Huynh-Feldt | .001 | 1.000 | .001 | 6.889 | .009 |
|  |  | Lower-bound | .001 | 1.000 | .001 | 6.889 | .009 |
|  | SKEL | Sphericity Assumed | .000 | 1 | .000 | 7.833 | .006 |
|  |  | Greenhouse-Geisser | .000 | 1.000 | .000 | 7.833 | .006 |
|  |  | Huynh-Feldt | .000 | 1.000 | .000 | 7.833 | .006 |
|  |  | Lower-bound | .000 | 1.000 | .000 | 7.833 | .006 |
|  | SLF | Sphericity Assumed | .000 | 1 | .000 | 2.835 | .094 |
|  |  | Greenhouse-Geisser | .000 | 1.000 | .000 | 2.835 | .094 |
|  |  | Huynh-Feldt | .000 | 1.000 | .000 | 2.835 | .094 |
|  |  | Lower-bound | .000 | 1.000 | .000 | 2.835 | .094 |
|  | UNC_pfc | Sphericity Assumed | .001 | 1 | .001 | 11.448 | .001 |
|  |  | Greenhouse-Geisser | .001 | 1.000 | .001 | 11.448 | .001 |
|  |  | Huynh-Feldt | .001 | 1.000 | .001 | 11.448 | .001 |
|  |  | Lower-bound | .001 | 1.000 | .001 | 11.448 | .001 |
| time * Treatment_4group | acc | Sphericity Assumed | .001 | 3 | .000 | 1.561 | .201 |
|  |  | Greenhouse-Geisser | .001 | 3.000 | .000 | 1.561 | .201 |
|  |  | Huynh-Feldt | .001 | 3.000 | .000 | 1.561 | .201 |
|  |  | Lower-bound | .001 | 3.000 | .000 | 1.561 | .201 |
|  | alic | Sphericity Assumed | .000 | 3 | 8.085E-5 | .688 | .560 |
|  |  | Greenhouse-Geisser | .000 | 3.000 | 8.085E-5 | .688 | .560 |
|  |  | Huynh-Feldt | .000 | 3.000 | 8.085E-5 | .688 | .560 |
|  |  | Lower-bound | .000 | 3.000 | 8.085E-5 | .688 | .560 |
|  | cc | Sphericity Assumed | .000 | 3 | 5.707E-5 | .355 | .786 |
|  |  | Greenhouse-Geisser | .000 | 3.000 | 5.707E-5 | .355 | .786 |
|  |  | Huynh-Feldt | .000 | 3.000 | 5.707E-5 | .355 | .786 |
|  |  | Lower-bound | .000 | 3.000 | 5.707E-5 | .355 | .786 |
|  | ec | Sphericity Assumed | .000 | 3 | 9.781E-5 | .916 | .434 |
|  |  | Greenhouse-Geisser | .000 | 3.000 | 9.781E-5 | .916 | .434 |
|  |  | Huynh-Feldt | .000 | 3.000 | 9.781E-5 | .916 | .434 |
|  |  | Lower-bound | .000 | 3.000 | 9.781E-5 | .916 | .434 |
|  | fMAJ | Sphericity Assumed | 5.695E-5 | 3 | 1.898E-5 | .207 | .891 |
|  |  | Greenhouse-Geisser | 5.695E-5 | 3.000 | 1.898E-5 | .207 | .891 |
|  |  | Huynh-Feldt | 5.695E-5 | 3.000 | 1.898E-5 | .207 | .891 |
|  |  | Lower-bound | 5.695E-5 | 3.000 | 1.898E-5 | .207 | .891 |
|  | fMIN | Sphericity Assumed | .001 | 3 | .000 | 1.764 | .156 |
|  |  | Greenhouse-Geisser | .001 | 3.000 | .000 | 1.764 | .156 |
|  |  | Huynh-Feldt | .001 | 3.000 | .000 | 1.764 | .156 |
|  |  | Lower-bound | .001 | 3.000 | .000 | 1.764 | .156 |
|  | FX | Sphericity Assumed | .005 | 3 | .002 | 5.606 | .001 |
|  |  | Greenhouse-Geisser | .005 | 3.000 | .002 | 5.606 | .001 |
|  |  | Huynh-Feldt | .005 | 3.000 | .002 | 5.606 | .001 |
|  |  | Lower-bound | .005 | 3.000 | .002 | 5.606 | .001 |
|  | gyrRect | Sphericity Assumed | .000 | 3 | 4.970E-5 | .360 | .782 |
|  |  | Greenhouse-Geisser | .000 | 3.000 | 4.970E-5 | .360 | .782 |
|  |  | Huynh-Feldt | .000 | 3.000 | 4.970E-5 | .360 | .782 |
|  |  | Lower-bound | .000 | 3.000 | 4.970E-5 | .360 | .782 |
|  | HIPP_st | Sphericity Assumed | .001 | 3 | .000 | .747 | .526 |
|  |  | Greenhouse-Geisser | .001 | 3.000 | .000 | .747 | .526 |
|  |  | Huynh-Feldt | .001 | 3.000 | .000 | .747 | .526 |
|  |  | Lower-bound | .001 | 3.000 | .000 | .747 | .526 |
|  | ILF_temp | Sphericity Assumed | .000 | 3 | 4.171E-5 | .321 | .810 |
|  |  | Greenhouse-Geisser | .000 | 3.000 | 4.171E-5 | .321 | .810 |
|  |  | Huynh-Feldt | .000 | 3.000 | 4.171E-5 | .321 | .810 |
|  |  | Lower-bound | .000 | 3.000 | 4.171E-5 | .321 | .810 |
|  | IFOF_ILF_occ | Sphericity Assumed | .001 | 3 | .000 | .966 | .410 |
|  |  | Greenhouse-Geisser | .001 | 3.000 | .000 | .966 | .410 |
|  |  | Huynh-Feldt | .001 | 3.000 | .000 | .966 | .410 |
|  |  | Lower-bound | .001 | 3.000 | .000 | .966 | .410 |
|  | IFOF_UNC | Sphericity Assumed | .000 | 3 | .000 | .674 | .569 |
|  |  | Greenhouse-Geisser | .000 | 3.000 | .000 | .674 | .569 |
|  |  | Huynh-Feldt | .000 | 3.000 | .000 | .674 | .569 |
|  |  | Lower-bound | .000 | 3.000 | .000 | .674 | .569 |
|  | PCC | Sphericity Assumed | .001 | 3 | .000 | .184 | .907 |
|  |  | Greenhouse-Geisser | .001 | 3.000 | .000 | .184 | .907 |
|  |  | Huynh-Feldt | .001 | 3.000 | .000 | .184 | .907 |
|  |  | Lower-bound | .001 | 3.000 | .000 | .184 | .907 |
|  | PLIC | Sphericity Assumed | .000 | 3 | .000 | .701 | .553 |
|  |  | Greenhouse-Geisser | .000 | 3.000 | .000 | .701 | .553 |
|  |  | Huynh-Feldt | .000 | 3.000 | .000 | .701 | .553 |
|  |  | Lower-bound | .000 | 3.000 | .000 | .701 | .553 |
|  | prefrontal | Sphericity Assumed | .000 | 3 | 4.778E-5 | 1.274 | .285 |
|  |  | Greenhouse-Geisser | .000 | 3.000 | 4.778E-5 | 1.274 | .285 |
|  |  | Huynh-Feldt | .000 | 3.000 | 4.778E-5 | 1.274 | .285 |
|  |  | Lower-bound | .000 | 3.000 | 4.778E-5 | 1.274 | .285 |
|  | reg1cc | Sphericity Assumed | 7.018E-5 | 3 | 2.339E-5 | .179 | .910 |
|  |  | Greenhouse-Geisser | 7.018E-5 | 3.000 | 2.339E-5 | .179 | .910 |
|  |  | Huynh-Feldt | 7.018E-5 | 3.000 | 2.339E-5 | .179 | .910 |
|  |  | Lower-bound | 7.018E-5 | 3.000 | 2.339E-5 | .179 | .910 |
|  | reg2cc | Sphericity Assumed | .002 | 3 | .001 | 1.215 | .306 |
|  |  | Greenhouse-Geisser | .002 | 3.000 | .001 | 1.215 | .306 |
|  |  | Huynh-Feldt | .002 | 3.000 | .001 | 1.215 | .306 |
|  |  | Lower-bound | .002 | 3.000 | .001 | 1.215 | .306 |
|  | reg3cc | Sphericity Assumed | .000 | 3 | 4.469E-5 | .083 | .969 |
|  |  | Greenhouse-Geisser | .000 | 3.000 | 4.469E-5 | .083 | .969 |
|  |  | Huynh-Feldt | .000 | 3.000 | 4.469E-5 | .083 | .969 |
|  |  | Lower-bound | .000 | 3.000 | 4.469E-5 | .083 | .969 |
|  | reg4cc | Sphericity Assumed | .001 | 3 | .000 | .409 | .747 |
|  |  | Greenhouse-Geisser | .001 | 3.000 | .000 | .409 | .747 |
|  |  | Huynh-Feldt | .001 | 3.000 | .000 | .409 | .747 |
|  |  | Lower-bound | .001 | 3.000 | .000 | .409 | .747 |
|  | reg5cc | Sphericity Assumed | .000 | 3 | 7.200E-5 | .708 | .548 |
|  |  | Greenhouse-Geisser | .000 | 3.000 | 7.200E-5 | .708 | .548 |
|  |  | Huynh-Feldt | .000 | 3.000 | 7.200E-5 | .708 | .548 |
|  |  | Lower-bound | .000 | 3.000 | 7.200E-5 | .708 | .548 |
|  | SCR | Sphericity Assumed | .000 | 3 | 6.252E-5 | .808 | .491 |
|  |  | Greenhouse-Geisser | .000 | 3.000 | 6.252E-5 | .808 | .491 |
|  |  | Huynh-Feldt | .000 | 3.000 | 6.252E-5 | .808 | .491 |
|  |  | Lower-bound | .000 | 3.000 | 6.252E-5 | .808 | .491 |
|  | SKEL | Sphericity Assumed | 6.491E-5 | 3 | 2.164E-5 | .595 | .619 |
|  |  | Greenhouse-Geisser | 6.491E-5 | 3.000 | 2.164E-5 | .595 | .619 |
|  |  | Huynh-Feldt | 6.491E-5 | 3.000 | 2.164E-5 | .595 | .619 |
|  |  | Lower-bound | 6.491E-5 | 3.000 | 2.164E-5 | .595 | .619 |
|  | SLF | Sphericity Assumed | .000 | 3 | 3.872E-5 | .740 | .530 |
|  |  | Greenhouse-Geisser | .000 | 3.000 | 3.872E-5 | .740 | .530 |
|  |  | Huynh-Feldt | .000 | 3.000 | 3.872E-5 | .740 | .530 |
|  |  | Lower-bound | .000 | 3.000 | 3.872E-5 | .740 | .530 |
|  | UNC_pfc | Sphericity Assumed | 8.249E-5 | 3 | 2.750E-5 | .251 | .861 |
|  |  | Greenhouse-Geisser | 8.249E-5 | 3.000 | 2.750E-5 | .251 | .861 |
|  |  | Huynh-Feldt | 8.249E-5 | 3.000 | 2.750E-5 | .251 | .861 |
|  |  | Lower-bound | 8.249E-5 | 3.000 | 2.750E-5 | .251 | .861 |
| Error(time) | acc | Sphericity Assumed | .025 | 170 | .000 |  |  |
|  |  | Greenhouse-Geisser | .025 | 170.000 | .000 |  |  |
|  |  | Huynh-Feldt | .025 | 170.000 | .000 |  |  |
|  |  | Lower-bound | .025 | 170.000 | .000 |  |  |
|  | alic | Sphericity Assumed | .020 | 170 | .000 |  |  |
|  |  | Greenhouse-Geisser | .020 | 170.000 | .000 |  |  |
|  |  | Huynh-Feldt | .020 | 170.000 | .000 |  |  |
|  |  | Lower-bound | .020 | 170.000 | .000 |  |  |
|  | cc | Sphericity Assumed | .027 | 170 | .000 |  |  |
|  |  | Greenhouse-Geisser | .027 | 170.000 | .000 |  |  |
|  |  | Huynh-Feldt | .027 | 170.000 | .000 |  |  |
|  |  | Lower-bound | .027 | 170.000 | .000 |  |  |
|  | ec | Sphericity Assumed | .018 | 170 | .000 |  |  |
|  |  | Greenhouse-Geisser | .018 | 170.000 | .000 |  |  |
|  |  | Huynh-Feldt | .018 | 170.000 | .000 |  |  |
|  |  | Lower-bound | .018 | 170.000 | .000 |  |  |
|  | fMAJ | Sphericity Assumed | .016 | 170 | 9.168E-5 |  |  |
|  |  | Greenhouse-Geisser | .016 | 170.000 | 9.168E-5 |  |  |
|  |  | Huynh-Feldt | .016 | 170.000 | 9.168E-5 |  |  |
|  |  | Lower-bound | .016 | 170.000 | 9.168E-5 |  |  |
|  | fMIN | Sphericity Assumed | .017 | 170 | .000 |  |  |
|  |  | Greenhouse-Geisser | .017 | 170.000 | .000 |  |  |
|  |  | Huynh-Feldt | .017 | 170.000 | .000 |  |  |
|  |  | Lower-bound | .017 | 170.000 | .000 |  |  |
|  | FX | Sphericity Assumed | .053 | 170 | .000 |  |  |
|  |  | Greenhouse-Geisser | .053 | 170.000 | .000 |  |  |
|  |  | Huynh-Feldt | .053 | 170.000 | .000 |  |  |
|  |  | Lower-bound | .053 | 170.000 | .000 |  |  |
|  | gyrRect | Sphericity Assumed | .023 | 170 | .000 |  |  |
|  |  | Greenhouse-Geisser | .023 | 170.000 | .000 |  |  |
|  |  | Huynh-Feldt | .023 | 170.000 | .000 |  |  |
|  |  | Lower-bound | .023 | 170.000 | .000 |  |  |
|  | HIPP_st | Sphericity Assumed | .080 | 170 | .000 |  |  |
|  |  | Greenhouse-Geisser | .080 | 170.000 | .000 |  |  |
|  |  | Huynh-Feldt | .080 | 170.000 | .000 |  |  |
|  |  | Lower-bound | .080 | 170.000 | .000 |  |  |
|  | ILF_temp | Sphericity Assumed | .022 | 170 | .000 |  |  |
|  |  | Greenhouse-Geisser | .022 | 170.000 | .000 |  |  |
|  |  | Huynh-Feldt | .022 | 170.000 | .000 |  |  |
|  |  | Lower-bound | .022 | 170.000 | .000 |  |  |
|  | IFOF_ILF_occ | Sphericity Assumed | .032 | 170 | .000 |  |  |
|  |  | Greenhouse-Geisser | .032 | 170.000 | .000 |  |  |
|  |  | Huynh-Feldt | .032 | 170.000 | .000 |  |  |
|  |  | Lower-bound | .032 | 170.000 | .000 |  |  |
|  | IFOF_UNC | Sphericity Assumed | .026 | 170 | .000 |  |  |
|  |  | Greenhouse-Geisser | .026 | 170.000 | .000 |  |  |
|  |  | Huynh-Feldt | .026 | 170.000 | .000 |  |  |
|  |  | Lower-bound | .026 | 170.000 | .000 |  |  |
|  | PCC | Sphericity Assumed | .259 | 170 | .002 |  |  |
|  |  | Greenhouse-Geisser | .259 | 170.000 | .002 |  |  |
|  |  | Huynh-Feldt | .259 | 170.000 | .002 |  |  |
|  |  | Lower-bound | .259 | 170.000 | .002 |  |  |
|  | PLIC | Sphericity Assumed | .028 | 170 | .000 |  |  |
|  |  | Greenhouse-Geisser | .028 | 170.000 | .000 |  |  |
|  |  | Huynh-Feldt | .028 | 170.000 | .000 |  |  |
|  |  | Lower-bound | .028 | 170.000 | .000 |  |  |
|  | prefrontal | Sphericity Assumed | .006 | 170 | 3.752E-5 |  |  |
|  |  | Greenhouse-Geisser | .006 | 170.000 | 3.752E-5 |  |  |
|  |  | Huynh-Feldt | .006 | 170.000 | 3.752E-5 |  |  |
|  |  | Lower-bound | .006 | 170.000 | 3.752E-5 |  |  |
|  | reg1cc | Sphericity Assumed | .022 | 170 | .000 |  |  |
|  |  | Greenhouse-Geisser | .022 | 170.000 | .000 |  |  |
|  |  | Huynh-Feldt | .022 | 170.000 | .000 |  |  |
|  |  | Lower-bound | .022 | 170.000 | .000 |  |  |
|  | reg2cc | Sphericity Assumed | .082 | 170 | .000 |  |  |
|  |  | Greenhouse-Geisser | .082 | 170.000 | .000 |  |  |
|  |  | Huynh-Feldt | .082 | 170.000 | .000 |  |  |
|  |  | Lower-bound | .082 | 170.000 | .000 |  |  |
|  | reg3cc | Sphericity Assumed | .091 | 170 | .001 |  |  |
|  |  | Greenhouse-Geisser | .091 | 170.000 | .001 |  |  |
|  |  | Huynh-Feldt | .091 | 170.000 | .001 |  |  |
|  |  | Lower-bound | .091 | 170.000 | .001 |  |  |
|  | reg4cc | Sphericity Assumed | .108 | 170 | .001 |  |  |
|  |  | Greenhouse-Geisser | .108 | 170.000 | .001 |  |  |
|  |  | Huynh-Feldt | .108 | 170.000 | .001 |  |  |
|  |  | Lower-bound | .108 | 170.000 | .001 |  |  |
|  | reg5cc | Sphericity Assumed | .017 | 170 | .000 |  |  |
|  |  | Greenhouse-Geisser | .017 | 170.000 | .000 |  |  |
|  |  | Huynh-Feldt | .017 | 170.000 | .000 |  |  |
|  |  | Lower-bound | .017 | 170.000 | .000 |  |  |
|  | SCR | Sphericity Assumed | .013 | 170 | 7.739E-5 |  |  |
|  |  | Greenhouse-Geisser | .013 | 170.000 | 7.739E-5 |  |  |
|  |  | Huynh-Feldt | .013 | 170.000 | 7.739E-5 |  |  |
|  |  | Lower-bound | .013 | 170.000 | 7.739E-5 |  |  |
|  | SKEL | Sphericity Assumed | .006 | 170 | 3.635E-5 |  |  |
|  |  | Greenhouse-Geisser | .006 | 170.000 | 3.635E-5 |  |  |
|  |  | Huynh-Feldt | .006 | 170.000 | 3.635E-5 |  |  |
|  |  | Lower-bound | .006 | 170.000 | 3.635E-5 |  |  |
|  | SLF | Sphericity Assumed | .009 | 170 | 5.235E-5 |  |  |
|  |  | Greenhouse-Geisser | .009 | 170.000 | 5.235E-5 |  |  |
|  |  | Huynh-Feldt | .009 | 170.000 | 5.235E-5 |  |  |
|  |  | Lower-bound | .009 | 170.000 | 5.235E-5 |  |  |
|  | UNC_pfc | Sphericity Assumed | .019 | 170 | .000 |  |  |
|  |  | Greenhouse-Geisser | .019 | 170.000 | .000 |  |  |
|  |  | Huynh-Feldt | .019 | 170.000 | .000 |  |  |
|  |  | Lower-bound | .019 | 170.000 | .000 |  |  |

**Supplementary Material 5**

**Table 5.** Result of the post-hoc pairwise comparison tests (p-values) of the repeated measures ANOVA, showing differences in diffusivity changes between four interventions in the fornix.

| Group | FA | RD | MD |
| --- | --- | --- | --- |
| Dance vs. Control | 0.000 | 0.000 | 0.003 |
| Dance vs. Walking | 0.002 | 0.018 | 0.071 |
| Dance vs. Walking + nutrition | 0.009 | 0.205 | 0.568 |
| Walking vs. Walking + nutrition | 0.872 | 0.417 | 0.307 |
| Control vs. Walking + nutrition | 0.356 | 0.070 | 0.038 |
| Control vs. Walking | 0.402 | 0.259 | 0.249 |

**Supplementary Material 6**


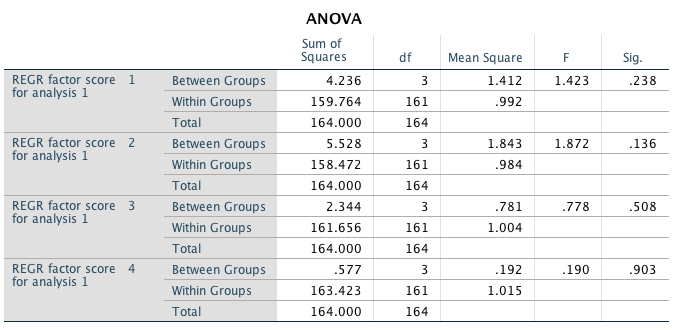
**Table 6.** One-way ANOVA on PCA values of the four cognitive domains at baseline.

REGR factor score 1: vocabulary, 2: fluid intelligence, 3: processing speed, 4: memory.

References

1. Raven, J. (1962). *Advanced Progressive Matrices: Sets 1 and 2.* London: H. K. Lewis.

2. Zachary, R. A. (1986). *Shipley Institute of Living Scale: Revised Manual.* Los Angeles: Western Psychological Services.

3. Ekstrom, R., French, J., Harman, H., & Dermen, D.(1976). *Manual for Kit of Factor-referenced Cognitive Tests.* Princeton: Educational Testing Service.

4. Bennett, G., Seashore, H., & Wesman, A.(1997) Differential Aptitude Test. *San Antonio: The Psychological Corporation*.

5. Wechsler, D. (1997). Wechsler Adult Intelligence Scale. Third. *San Antonio: The Psychological Corporation.*

6. Salthouse, T. A., & Babcock, R. L. (1991). Decomposing adult age differences in working memory. *Dev Psychol. American Psychological Association, 27,* 763–776. doi:10.1037/0012-1649.27.5.763

7. Salthouse, T. A., Fristoe, N., & Rhee, S. H. (1996). How localized are age-related effects on neuropsychological measures? *Neuropsychology,* 10, 272–285.

8. Woodcock, R. W., & Johnson, M. B. (1990). Woodcock-Johnson Psycho-Educational Battery-Revised. *Allen, TX*: DLM.

9. Salthouse, T. A. (1993). Speed and knowledge as determinants of adult age differences in verbal tasks. *Journals Gerontology, 48,* 29-36.
